# Supplementary material for: Dual targeting of lymphocyte homing and retention through α4β7 and αEβ7 inhibition in inflammatory bowel disease
Source: Cell Rep Med. 2021 Aug 17;2(8):100381. doi: 10.1016/j.xcrm.2021.100381 (PMC8385326; doi:10.1016/j.xcrm.2021.100381)
Supplement: Document S1. Figures S1–S7, Tables S1 and S2, and Data S1 [file mmc1.pdf]

Cell Reports Medicine, Volume *2*

Supplemental information

Dual targeting of lymphocyte homing and retention through α4β7 and αEβ7 inhibition in inflammatory bowel disease

Bingbing Dai, Jason A. Hackney, Ryan Ichikawa, Allen Nguyen, Justin Elstrott, Luz D. Orozco, Kai-Hui Sun, Zora Modrusan, Alvin Gogineni, Alexis Scherl, John Gubatan, Aida Habtezion, Monika Deswal, Ma Somsouk, William A. Faubion, Akiko Chai, Zaineb Sharafali, Azra Hassanali, Young S. Oh, Swati Tole, Jacqueline McBride, Mary E. Keir, and Tangsheng Yi

Supplemental Figure and Figure legend

Figure S1

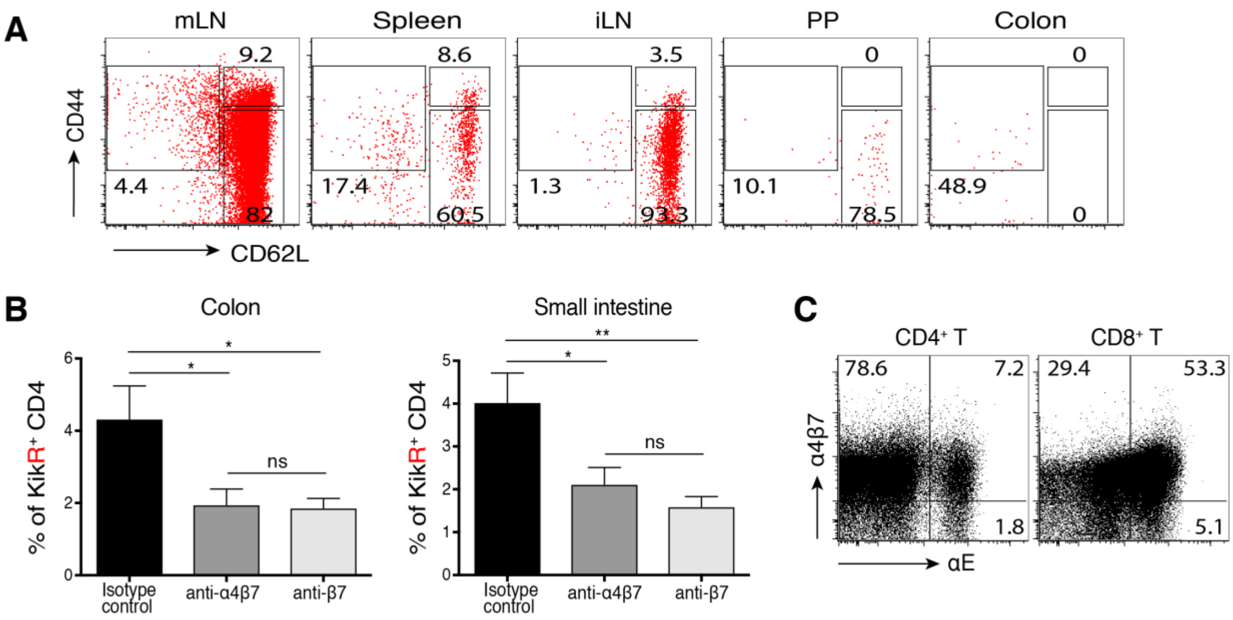

Figure S1. Tissue distribution and surface integrin expression of T cells following surgical photoconversion. Related to Figure 1. (**A**) Expression of CD44 and CD62L on $KikR^+$ $CD8^+$ T cells from lymphoid and gut tissues 16 hours after mLN photoconversion. (**B**) Frequency of photo-converted $KikR^+$ cells among $CD45^+TCR\beta^+CD4^+$ T cells in colon (**left**) and small intestine (**right**). KikGR transgenic mice were treated with isotype or anti-integrin antibodies followed by surgical photoconversion of mLN. Small intestine and colon tissue were taken 16 hours post-photoconversion for flow cytometry analysis. Bar graph shows mean ± standard error (SEM). n=6-8 mice combined from 2 independent experiments where *=p<0.05, **=p<0.01, ***=p<0.001 by one-way ANOVA with Tukey's multiple comparison post-test. (**C**) Flow cytometry analysis of α4β7 and αE expression on gated $CD4^+$ or $CD8^+$ T cells from mLN. One representative plot is shown of three mice analyzed.

Figure S2

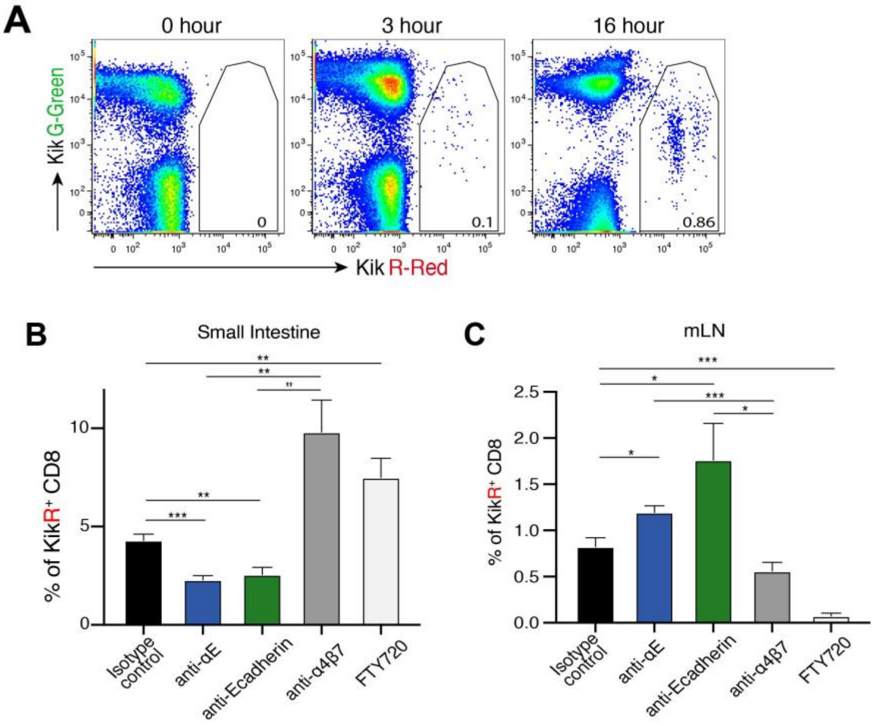

Figure S2. T cells photoconverted in the small intestine emigrate to the draining lymph node. Related to Figure 4. KikGR$^+$ OT-1 cells were adoptively transferred into WT mice, then recipient mice were orally gavaged with cholera toxin:OVA. Three days post immunization, isotype or anti-integrin antibodies were administered followed by surgical photoconversion of a segment of the small intestine. After photo-conversion, small intestine and mLN were collected and cells were isolated and stained for flow cytometry analysis. (**A**) Representative FACS plot of mLN at 0, 3 and 16 hours following small intestine photostamping. (**B**) Percentage of KikR$^+$ OT-1 cells in the small intestine (**B**) and mLN (**C**) 16 hours after photoconversion. Bar graph shows mean ± (SEM) of 6-8 animals combined from two to three independent experiments. One-way ANOVA with Tukey's multiple comparison post-test was used to compare statistical significance, *=p<0.05, **=p<0.01, ***=p<0.001, ****=p<0.0001

Figure S3

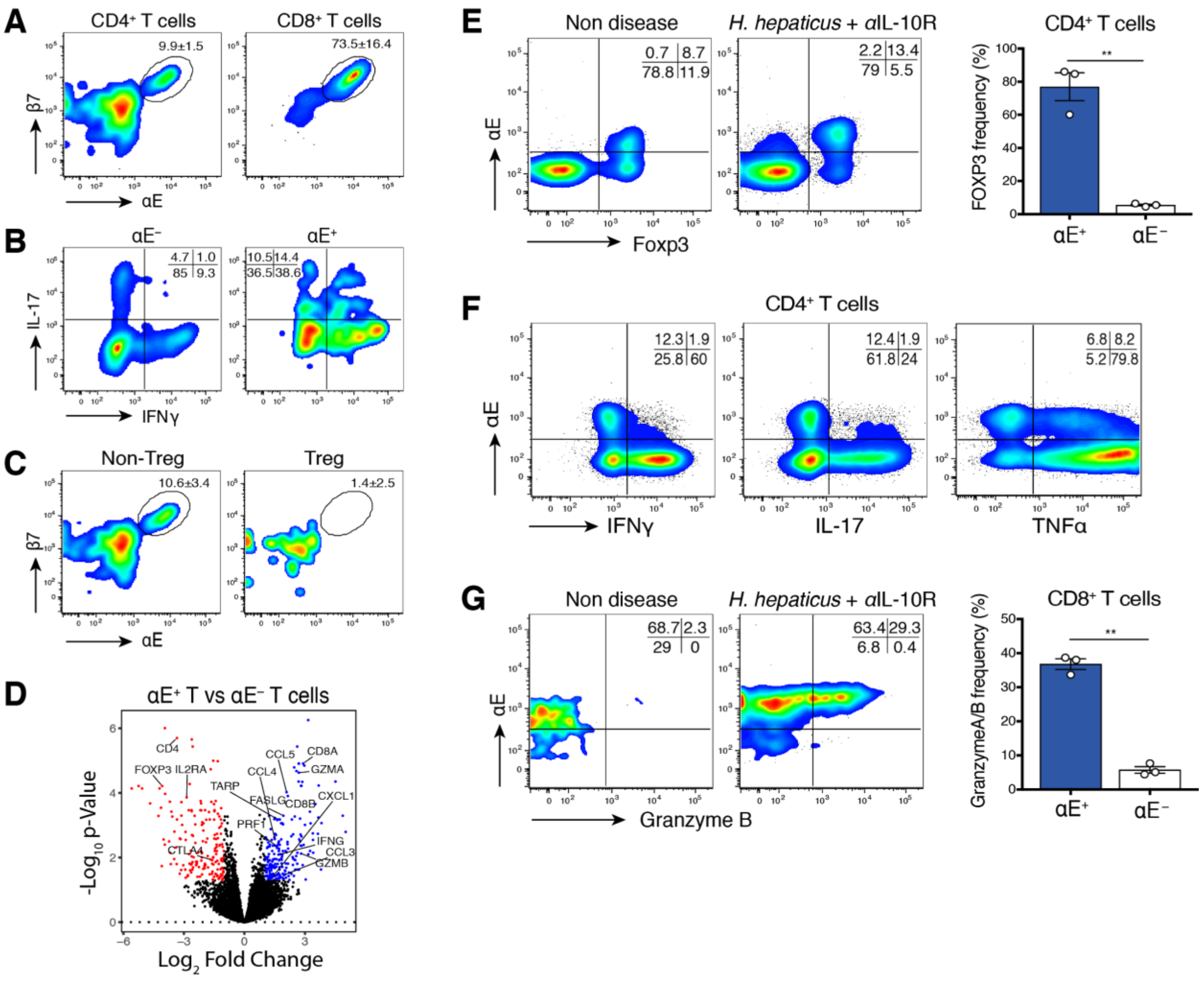

Figure S3. Proinflammatory state of αE^{+} CD4^{+} human T cells is not observed in mouse αEβ7^{+} CD4^{+} T cells. Related to Figure 5. T cells isolated from colonic tissue were evaluated for integrin expression and inflammatory phenotype. **(A)** αE and β7 expression on colonic CD4^{+} and CD8^{+} T cells. Data is shown from a representative healthy colonic biopsy. (**B**) *Ex vivo* stimulation of colonic T cells induced IL-17 and IFN-γ production cells in αE^{+} or αE^{-} CD4^{+} T cells from a UC patient. (**C**) αE and β7 expression in colonic CD4^{+}Foxp3^{+}CD25^{+}CD127^{low} cells (Treg) and CD4^{+} T cells from a healthy subject. (**D**) Volcano plot showing gene expression in sorted αE^{+}TCRβ^{+} T cells vs αE^{-} TCRβ^{+} T cells from colonic resections (ulcerative colitis patients, n=4 and diverticulitis (non-IBD), n=4). (**E**) Similar phenotyping of mouse colonic T cells showing αE and Foxp3 expression in LP CD4^{+} T cells isolated from naive or colitic mice treated with *H. hepaticus* and anti-IL-10R blockade (left). Right panel, frequency of Foxp3^{+} regulatory T

cells among αE^+ $CD4^+$ T cells vs αE^- $CD4^+$ T cells isolated from colitic mice. Each dot represents an individual mouse and data is shown as mean ± SEM. (**F**) *Ex vivo* stimulation of colonic LP T cells from colitic mice treated with *H. hepaticus* and anti-IL-10R blockade induced intracellular cytokine expression. One representative flow cytometry pattern from four colitic animals in each group. (**G**) Left, αE and intracellular granzyme B expression in $CD8^+$ T cells isolated from colon LP in colitic mice treated with *H. hepaticus* and anti-IL-10R blockade. Right, frequency of granzyme A and B double positive cells among αE^+ or αE^- $CD8^+$ T cells from colitic mice. Each dot represents an individual mouse and data is shown as mean ± SEM. Data are representative of three independent experiments. Unpaired Student's t-test was used to compare statistical significance, *=$p<0.05$, **=$p<0.01$, ***=$p<0.001$, ****=$p<0.0001$

Figure S4

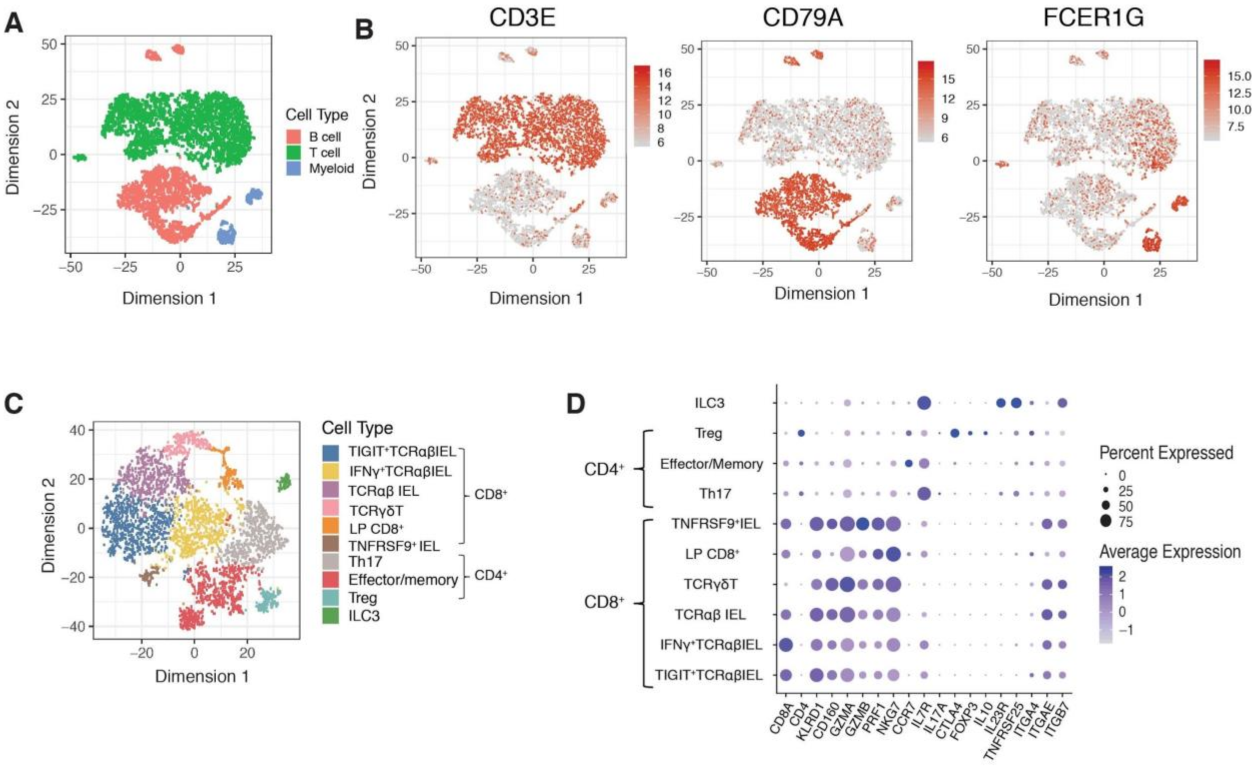

Figure S4. Clustering of human immune cells from colon. Related to Figure 5. (**A**) CD45^{+} immune cell census is shown as t-Stochastic Neighborhood Embedding (t-SNE) of cells colored by cell subsets. (**B**) Expression of CD3E, CD79A, and FCER1G in the corresponding cell population. (**C**) T cell census is shown as t-SNE of cells colored by cell subsets. (**D**) T cell subsets and their markers. Shown are fractions of expression (dot size) and mean expression level in expressing cells (dot color) of selected marker genes (columns) across subsets (rows).

Figure S5

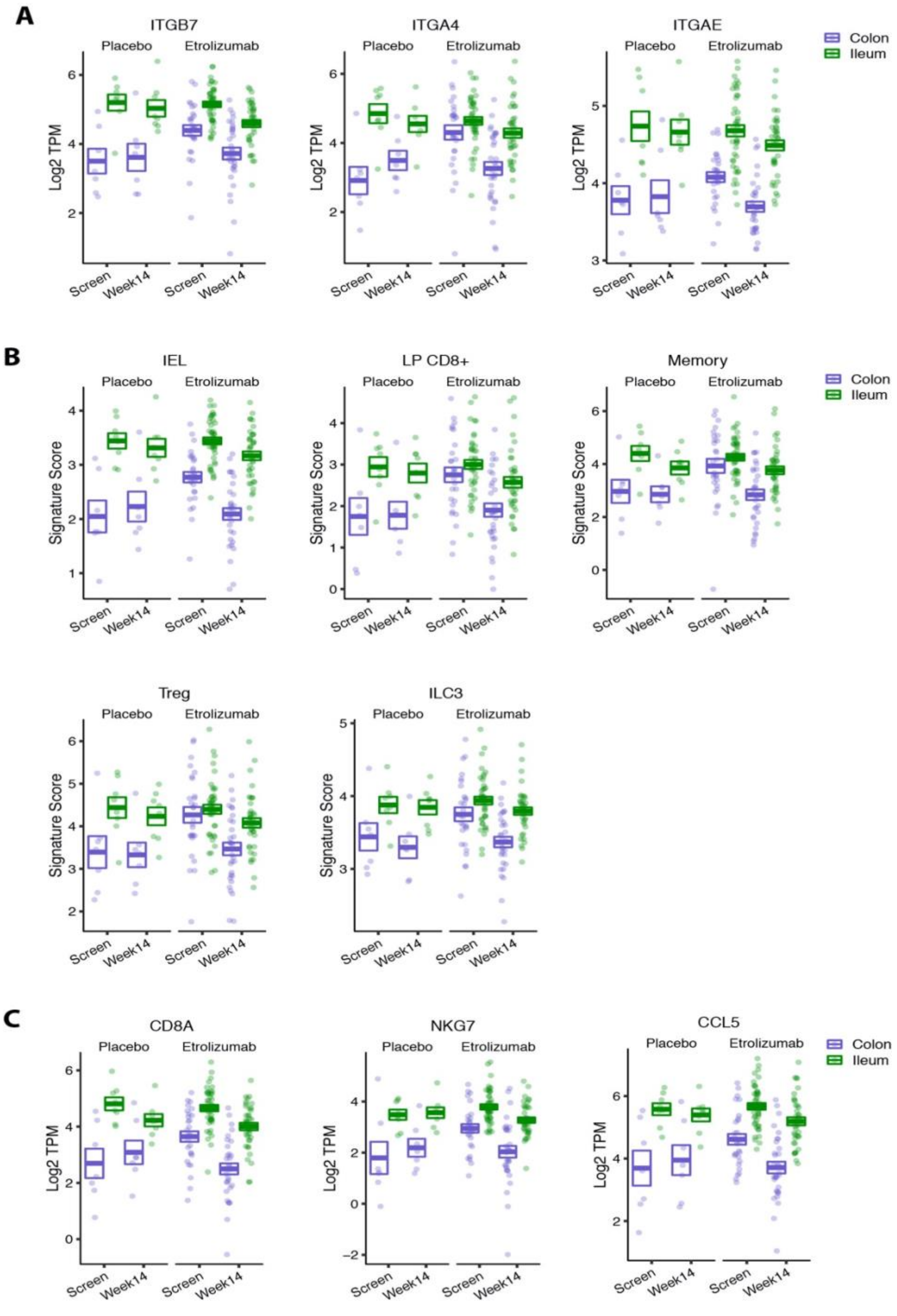

Figure S5. Etrolizumab treatment significantly reduces expression of genes associated with CD8$^+$ cytotoxic IELs. Related to Figure 6. Ileal or colonic biopsies were taken prior to treatment and at 14 weeks post-treatment in

a randomized placebo-controlled trial of etrolizumab (anti-β7 integrin) in patients with moderately to severely active CD (A) Expression of integrin genes at 14 weeks post-etrolizumab or placebo treatment. Each dot is an individual patient. Points are colored by sampling location (colon in blue, ileum in green). Boxes show the standard error, with the middle bar showing the group mean value. (B, C) Expression of the signature gene sets of indicated T cell subtype gene sets (B) and the cytotoxic IEL-specific genes (C) at week 14 in patients treated with etrolizumab or placebo. Each dot represents an individual patient sample. Points are colored by sampling location (colon in blue, ileum in green). Boxes show the standard error, with the middle bar showing the group mean value.

Figure S6

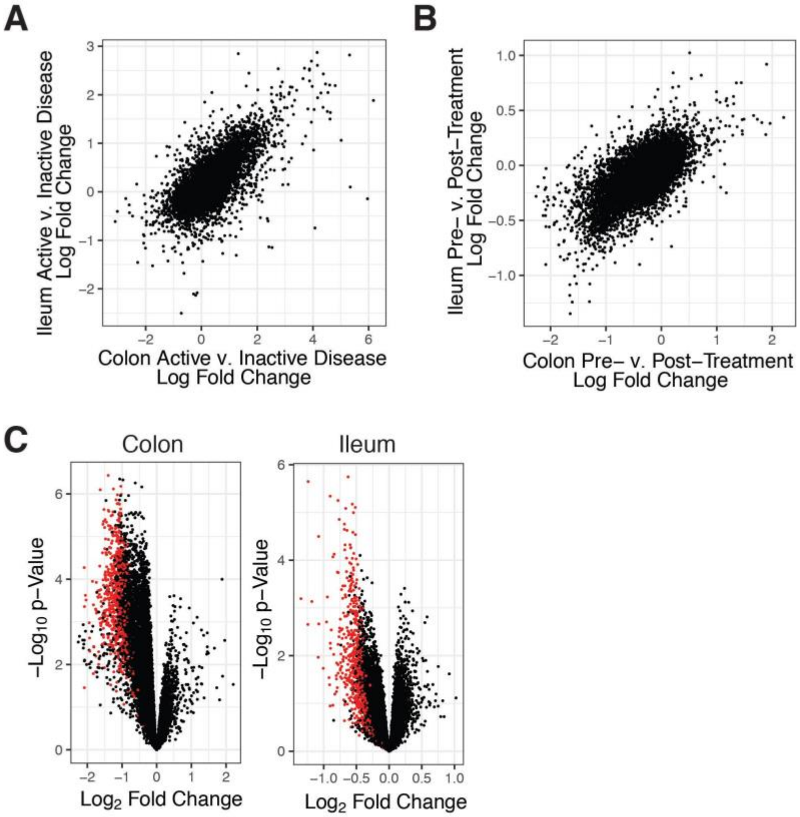

Figure S6. Etrolizumab treatment reduces inflammatory gene expression in both the colon and the ileum. Related to Figure 6. Scatter plots showing the log2 fold changes between samples taken from bowel segments with active vs inactive disease in either colon or ileum (**A**) or the log2 fold changes between samples taken from post-induction vs screening samples from etrolizumab-treated patients in either colon or ileum (**B**). (**C**) Volcano plot showing the log2 fold change and p-value of each gene comparing baseline to week 14 samples in etrolizumab-treated patients in either colon (left panel) or terminal ileum (right panel). Each point represents a gene, genes with a >1.5 fold change at an FDR < 0.05 in the pooled analysis combining the colon and ileum are shown in red.

Figure S7

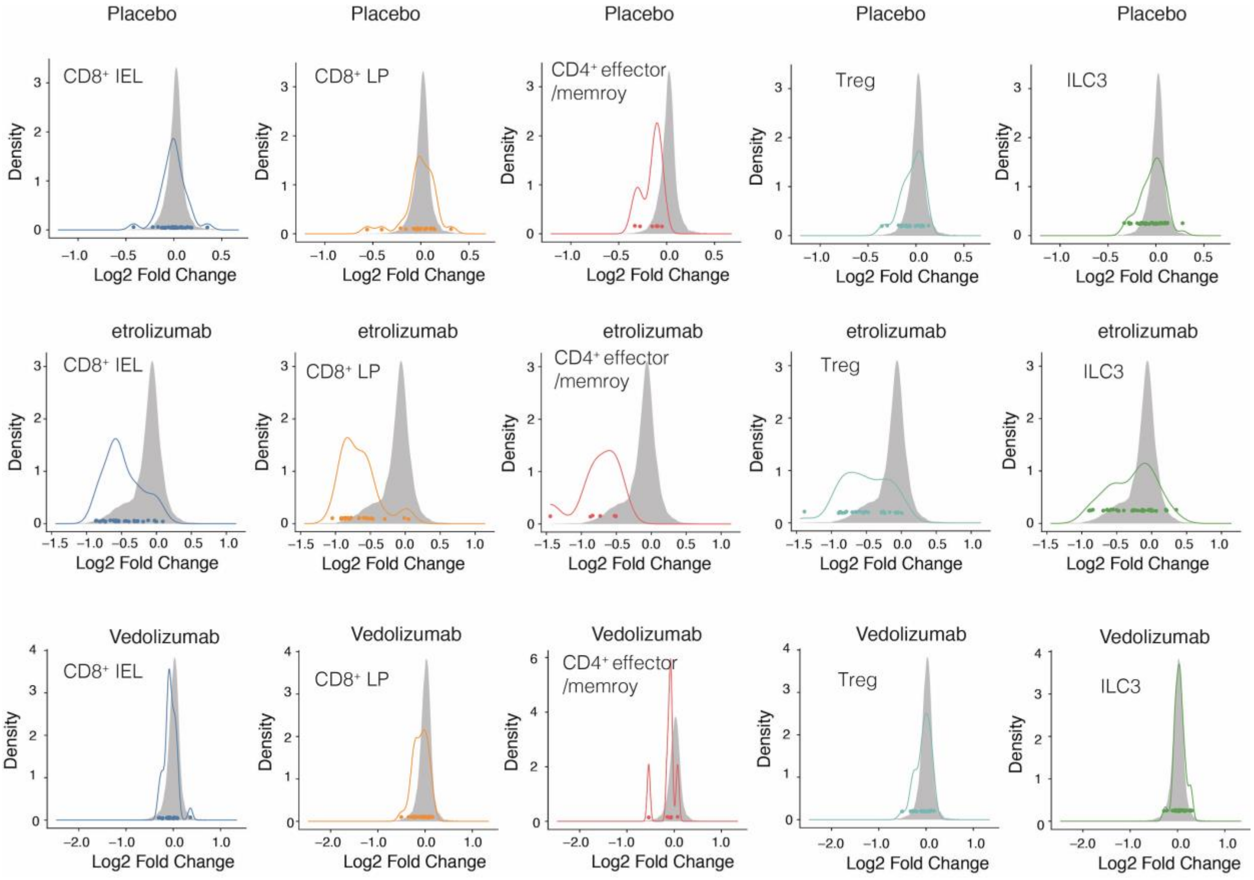

Figure S7. Etrolizumab, but not Vedolizumab or placebo, significantly reduces the gene set associated with CD8$^+$ IELs and CD8$^+$ LP populations. Related to Figure 6. Density plots of the slope of change in gene expression between baseline and post-treatment biopsies cohorts for each gene. Etrolizumab and placebo post-treatment biopsies were taken at week 14, while vedolizumab post-treatment biopsies were taken at week 12. The background distribution of all fold changes is shown in grey and the individual fold changes for genes in each T cell signature found to be downregulated in the placebo, vedolizumab or etrolizumab-treated patients are shown with solid points. The density distribution of these points are shown with solid lines.

Supplemental file 1. Heatmap of genes specific to each T cell subset. Each row is a gene, each column is a cell. T cell types are indicated across the top x-axis. Related to Figure 5.

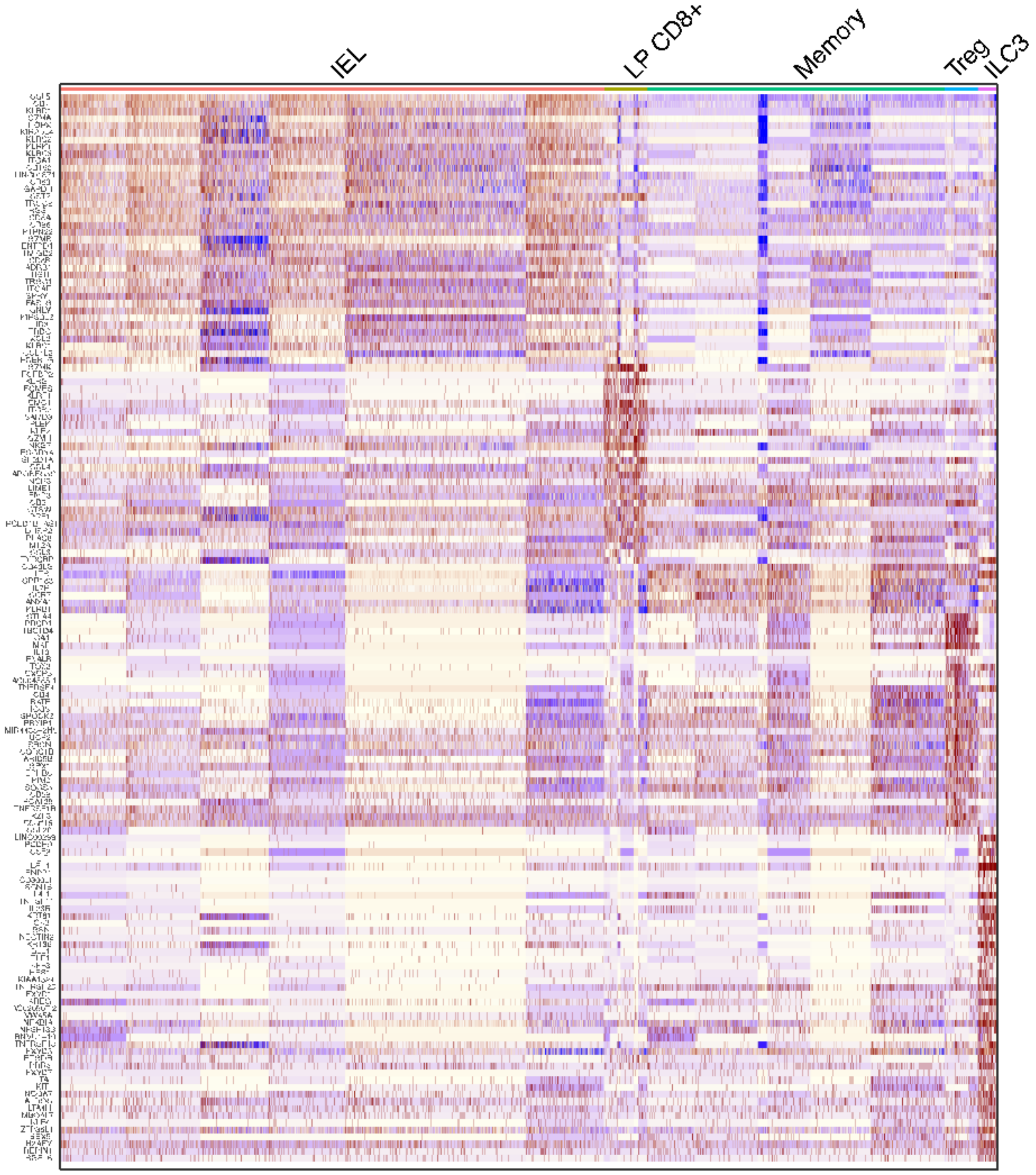

Table S1. Patient characteristics from observational cohorts. Related to Figure 5.

Cohort	Gender	Age	Diagnosis
Mayo Clinic	F/F	59	diverticulitis
	F/F	44	UC
	M/M	23	UC
	M/M	25	UC
	F/F	35	UC
	M/M	58	diverticulitis
	F/F	68	diverticulitis
	M/M	37	UC
Stanford	M/M	65	Healthy control
	F/F	58	UC
	F/F	56	UC
	F/F	42	CD
	F/F	38	CD
UCSF	F/F	32	Healthy control
	M/M	55	Healthy control
	F/F	34	Healthy control
	M/M	62	Healthy control
	M/M	38	Healthy control
	F/F	56	Healthy control
	M/M	26	Healthy control
	M/M	47	Healthy control
	M/M	68	Healthy control
	M/M	39	Healthy control

Table S2. Summary statistics for moderate to severe CD patients enrolled in etrolizumab clinical study with RNA sequencing data. Related to Figure 6.

**All subjects**	**Placebo (n=45)**		**Etrolizumab (n=172)**	
Biopsy location	colon (n=22)	ileum (n=23)	colon (n=70)	ileum (n=102)
Gender, female/male	9F / 13M	15F / 8M	35F / 35M	54F / 48M
Age in years, median (range)	31.5 (18-63)	39.0 (24-73)	35.0 (19-79)	42.0 (19-67)
SES-CD, median (range)	19.5 (6-44)	17.0 (5-35)	21.0 (9-38)	15.0 (5-50)
Disease extent Colonic (C), colonic+ileal (IC), ileal (I)	11C, 9IC, 2I	2C, 15IC, 6I	36C, 30IC, 4I	9C, 60IC, 33I
**Subjects with paired measures of active disease segments**	**Placebo (n=15)**		**Etrolizumab (n=71)**	
Biopsy location	colon (n=7)	ileum (n=8)	colon (n=28)	ileum (n=43)
Gender, female/male	6F / 1M	5F / 3M	18F / 10M	28F/15M
Age in years, median (range)	38.0 (27-51)	36.5 (24-62)	38.0 (19-79)	42.0 (19-67)
SES-CD, median (range)	19.0 (15-44)	14.5 (7-35)	21.0 (11-37)	15.0 (7-44)
Disease extent Colonic (C), colonic+ileal (IC), ileal (I)	4C, 3IC, 0I	0C, 5IC, 3I	16C, 12IC, 0I	0C, 27IC, 16I
