# Supplementary material for: Dual targeting of lymphocyte homing and retention through α4β7 and αEβ7 inhibition in inflammatory bowel disease
Source: Cell Rep Med. 2021 Aug 17;2(8):100381. doi: 10.1016/j.xcrm.2021.100381 (PMC8385326; doi:10.1016/j.xcrm.2021.100381)
Supplement: Document S2. Article plus supplemental information [file mmc5.pdf]

Cell Reports Medicine

Article

Dual targeting of lymphocyte homing and retention through α4β7 and αEβ7 inhibition in inflammatory bowel disease

Graphical abstract

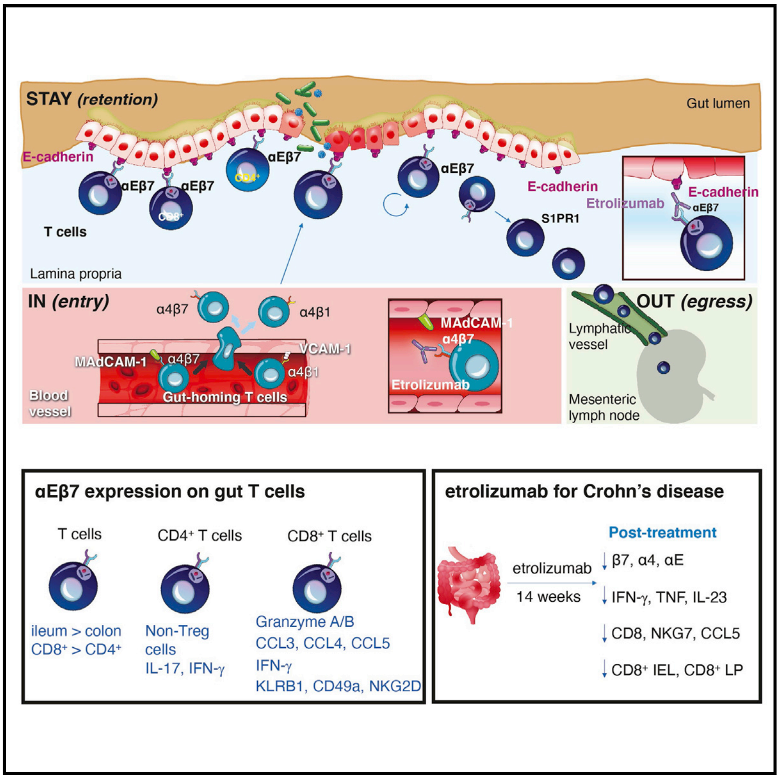

Highlights

- Blockade of α4β7 and αEβ7 reduces CD8^{+} T cells in the gut mucosa more than α4β7 alone
- Anti-αEβ7 or -E-cadherin reduces retention and increases egress of T cells in the gut
- αEβ7^{+} intestinal T cells are proinflammatory and have little to no regulatory markers
- Etrolizumab reduces mucosal inflammatory T cell genes in patients with Crohn disease

Authors

Bingbing Dai, Jason A. Hackney, Ryan Ichikawa, ..., Jacqueline McBride, Mary E. Keir, Tangsheng Yi

Correspondence

keir.mary@gene.com (M.E.K.),
yi.tangsheng@gene.com (T.Y.)

In brief

Dai et al. demonstrate the cooperative roles α4β7 and αEβ7 integrins have in CD8^{+} T cell accumulation in the gut mucosa in a mouse model of trafficking. Intestinal biopsies from patients with Crohn disease that has been treated with etrolizumab (anti-β7) show treatment-specific reductions in gene expression associated with CD8^{+} cytotoxic T cells.

Dai et al., 2021, Cell Reports Medicine *2*, 100381
August 17, 2021 © 2021 The Authors.
https://doi.org/10.1016/j.xcrm.2021.100381

CellPress

Cell Reports Medicine

CellPress
OPEN ACCESS

Article

Dual targeting of lymphocyte homing and retention through α4β7 and αEβ7 inhibition in inflammatory bowel disease

Bingbing Dai,[1,12] Jason A. Hackney,[4,12] Ryan Ichikawa,[3] Allen Nguyen,[4] Justin Elstrott,[5] Luz D. Orozco,[2] Kai-Hui Sun,[6] Zora Modrusan,[6] Alvin Gogineni,[5] Alexis Scherl,[7] John Gubatan,[8] Aida Habtezion,[8] Monika Deswal,[9] Ma Somsouk,[9] William A. Faubion,[10] Akiko Chai,[11] Zaineb Sharafali,[11] Azra Hassanali,[11] Young S. Oh,[11] Swati Tole,[11] Jacqueline McBride,[4] Mary E. Keir,[3,*] and Tangsheng Yi[1,13,*]

[1]Departments of Immunology Discovery, Genentech, Inc. 1 DNA Way, South San Francisco, CA 94080, USA
[2]Bioinformatics, Genentech, Inc. 1 DNA Way, South San Francisco, CA 94080, USA
[3]Biomarker Discovery OMNI, Genentech, Inc. 1 DNA Way, South San Francisco, CA 94080, USA
[4]OMNI Biomarker Development, Genentech, Inc. 1 DNA Way, South San Francisco, CA 94080, USA
[5]Biomedical Imaging, Genentech, Inc. 1 DNA Way, South San Francisco, CA 94080, USA
[6]Molecular Biology, Genentech, Inc. 1 DNA Way, South San Francisco, CA 94080, USA
[7]Pathology, Genentech, Inc. 1 DNA Way, South San Francisco, CA 94080, USA
[8]Division of Gastroenterology and Hepatology, Department of Medicine, Stanford University School of Medicine, Stanford, CA 94305, USA
[9]University of California, San Francisco (UCSF), San Francisco, CA 94143, USA
[10]Department of Medicine, Division of Gastroenterology and Hepatology, Mayo Clinic, Rochester, MN 55905, USA
[11]Product Development, Genentech, Inc. 1 DNA Way, South San Francisco, CA 94080, USA
[12]These authors contributed equally
[13]Lead contact
*Correspondence: keir.mary@gene.com (M.E.K.), yi.tangsheng@gene.com (T.Y.)
https://doi.org/10.1016/j.xcrm.2021.100381

SUMMARY

Anti-integrins are therapeutically effective for inflammatory bowel disease, yet the relative contribution of α4β7 and αEβ7 to gut lymphocyte trafficking is not fully elucidated. Here, we evaluate the effect of α4β7 and αEβ7 blockade using a combination of murine models of gut trafficking and longitudinal gene expression analysis in etrolizumab-treated patients with Crohn's disease (CD). Dual blockade of α4β7 and αEβ7 reduces $CD8^+$ T cell accumulation in the gut to a greater extent than blockade of either integrin alone. Anti-αEβ7 reduces epithelial:T cell interactions and promotes egress of activated T cells from the mucosa into lymphatics. Inflammatory gene expression is greater in human intestinal $\alpha E\beta 7^+$ T cells. Etrolizumab-treated patients with CD display a treatment-specific reduction in inflammatory and cytotoxic intraepithelial lymphocytes (IEL) genes. Concurrent blockade of α4β7 and αEβ7 promotes reduction of cytotoxic IELs and inflammatory T cells in the gut mucosa through a stepwise inhibition of intestinal tissue entry and retention.

INTRODUCTION

To mount an efficient immune response, lymphocytes travel between secondary lymphoid organs and mucosal tissues to enable antigen recognition, leading to activation and expansion. The gastrointestinal tract harbors a high antigenic load derived from food and microbiota and is a highly dynamic compartment in terms of immune cell movement.[1] In the mesenteric lymph node (mLN), activated T lymphocytes are imprinted in a specialized microenvironment that results in increased expression of integrin α4β7 and CCR9.[2,3] Elevated α4β7 enables T cells to have an increased capacity to adhere to mucosal addressin cell adhesion molecule 1 (MAdCAM-1) on endothelial venules in the lamina propria (LP).[4] Within the LP, T cells further upregulate αE integrin expression in response to the transforming growth factor β (TGF-β)-rich environment.[5,6] E-cadherin expressed by intestinal epithelial cells serves as a high-affinity ligand for αEβ7.[7–9] αEβ7 is also expressed on a subset of dendritic cells in both mouse and human and are involved in T cell differentiation and imprinting of homing receptors.[10] Gut-resident T cells also express CD69 to repress S1PR1-dependent tissue egress into lymphatics through CD69:S1P1 complex formation, another mechanism of lymphocyte retention in tissue.[11,12]

Inflammatory bowel disease (IBD) encompasses both ulcerative colitis (UC) and Crohn's disease (CD) and is characterized by aberrant inflammatory responses in the gastrointestinal (GI) tract.[13–15] Patients with IBD have increased activated lymphocytes in gut tissues, and anti-integrin therapies are efficacious.[15,16] Natalizumab, which blocks the α4 subunit of the α4β7 and α4β1 integrin heterodimers, was the first anti-integrin antibody approved for use in CD in 2004.[17] Vedolizumab is an anti-integrin monoclonal antibody that blocks the α4β7

This is an open access article under the CC BY license (http://creativecommons.org/licenses/by/4.0/).

heterodimer while sparing α4β1 integrin and is approved for both UC and CD.[18,19] Etrolizumab,[20–23] an investigational monoclonal antibody that blocks the β7 subunit of both α4β7 and αEβ7, is currently under development for CD.[24]

Despite the potential for gut-homing cells to be differentially affected by the integrin heterodimers targeted by these therapeutics, the relative contribution of α4β7 and αEβ7 in lymphocyte homing and retention is not yet fully understood.[25] Here, we established a surgical photoconversion system to investigate the integrin dependencies of T cell homing and retention in the gut and tissue egress to the mLN in a mouse model. Single-cell RNA sequencing (RNA-seq) and flow cytometry show that human inflammatory intestinal $CD8^+$ T cell subsets, and not regulatory T cells, have high expression of αEβ7. Consistent with that, we demonstrate a significant reduction of genes associated with cytotoxic IELs in patients with CD enrolled in a placebo-controlled study of etrolizumab (ClinicalTrials.gov: NCT02394028), which showed efficacy in an exploratory subcohort analysis.[26] Taken together, these mechanistic mouse model studies and human clinical studies suggest that targeting β7 integrin will effectively reduce inflammatory $CD8^+$ T cells and intestinal inflammation through dual effects on lymphocyte homing and retention.

RESULTS

Migration of $CD8^+$ T cells from mLN to gut mucosa is additively inhibited by combined blockade of α4β7 and αEβ7

To evaluate the role of integrins in T cell trafficking from mLN into the gut mucosa, we established a surgical photoconversion procedure in the KikGR transgenic mouse model.[27] KikGR protein changes color from green to red fluorescence upon exposure to violet light, which in these experiments was focused specifically on the mLN, thus "stamping" cells with red fluorescence to enable tracking their migration out of the mLN. As a proof-of-concept of this model, we evaluated steady-state mucosal immune cell trafficking from the mLN of control or FTY720 (a S1PR1 agonist)-dosed animals after photoconversion. Lymphocyte egress and re-distribution to various tissues was analyzed 16 h after photoconversion. Because of the critical role of S1PR1 in T cell egress from lymph nodes, most $KikR^+$ photo-stamped T cells are confined to the mLN of FTY720-treated animals (Figure 1A). In contrast, dynamic egress of mLN T cells was observed in vehicle-treated animals, with re-distribution of photo-stamped $KikR^+$ T cells to secondary lymphoid organs, including the spleen, inguinal LN, and Peyer's patches, as well as to the small intestine and colon (Figure 1A). Photo-stamped cells that migrated to the secondary lymphoid organs were mostly $CD62L^{high}CD44^{low}$ naive T cells, whereas T cells that had migrated into the colon LP were predominantly $CD44^{hi}$ effector/memory T cells (Figure S1A).

After the successful proof-of-concept study, this model was used to evaluate anti-α4β7 and anti-β7 effects on T cell trafficking between the mLN and intestine. Anti-α4β7 blockade reduced the frequency of migrated $KikR^+$ $CD4^+$ and $CD8^+$ T cells by 50% in the LP compartment of the colon and small intestine (Figures 1B–1D and S1B). Anti-β7 antibody blockade, which blocks both α4β7 and αEβ7, showed greater inhibition of $CD8^+$ T cells in comparison with that of $CD4^+$ T cells (Figures 1B–1D and S1B). Although most of the mLN $CD4^+$ and $CD8^+$ T cells express α4β7, αE expression is found on more than 60% of $CD8^+$ T cells but less than 10% of $CD4^+$ T cells (Figure S1C). Therefore, increased inhibition of $CD8^+$ T cells by anti-β7 is likely due to the differential expression of αE integrin between $CD8^+$ and $CD4^+$ T cells.

An anti-αE blocking antibody, which only binds to the αEβ7 heterodimer, was next used to further ascertain the individual contribution of α4β7 and αEβ7 in $CD8^+$ T cell migration from mLN to the gut.[28] Treatment with anti-αE blocking antibody significantly reduced photo-stamped $KikR^+$ $CD8^+$ T cells in the colon and small intestine as compared with control antibody (Figures 1E and 1F). As was previously observed with the β7 blockade, combined blockade of α4β7 and αEβ7 further reduced $KikR^+$ $CD8^+$ T cells ~50% more than blockade of either α4β7 or αEβ7 alone (Figures 1E and 1F). Taken together, our data suggest that blockade of α4β7 and αEβ7 has an additive effect in reducing $CD8^+$ T cell trafficking to the small and large intestine.

Combined blockade of α4β7 and αEβ7 reduced trafficking of antigen-specific T cells in comparison with blockade of α4β7 or αEβ7 alone

To test the role of α4β7 and αEβ7 in antigen-dependent T cell migration to the intestinal LP and epithelium, we used an oral antigen-challenge model to elicit an immune response in the small intestine.[29] Ovalbumin (OVA)-specific T cell receptor (TCR) transgenic MHC I-restricted (OT-1) T cells were adoptively transferred into wild-type (WT) mice before oral immunization with OVA and cholera toxin (CT:OVA). Anti-integrin antibodies were administered to the recipient mice 3 days after CT:OVA immunization when activated T cells began to migrate from the mLN to the small intestine. Treatment with anti-α4β7 or anti-αE blockade led to a reduction in OT-1 T cells in both the LP and intraepithelial compartments, whereas the combination of these blocking antibodies resulted in further reduction of OT-1 T cells in both compartments (Figures 2A and 2B). In this model, blockade of both α4β7 and αEβ7 leads to a greater reduction of antigen-specific $CD8^+$ T cells in comparison with blockade of either α4β7 or αEβ7 alone.

Blockade of αEβ7 or E-cadherin diminishes $CD8^+$ T cell interaction with intestinal epithelium

We next asked whether blockade of E-cadherin, the only known ligand of αEβ7, also regulates $CD8^+$ T cell accumulation similarly to anti-αE blockade. A blocking antibody against E-cadherin was used to disrupt E-cadherin:αEβ7 interactions after CT:OVA oral immunization.[28] OT-1 $CD8^+$ T cell accumulation in the intestinal mucosa was similarly reduced by anti-E-cadherin or anti-αE (Figure 3A), confirming that E-cadherin:αEβ7 interaction is critical for T cell accumulation in the gut epithelium.

T cells travel along and through the mesh-like structure of the basement membrane, an extracellular matrix composed of collagen and laminin, and interact with intestinal epithelial cells.[30,31] E-cadherin protein is found on the basolateral side of the intestinal epithelium (Figure 3B; Video S1). Activated OT-1 T cells expressing red fluorescent protein (OT-1-tdTomato)

CellPress
OPEN ACCESS

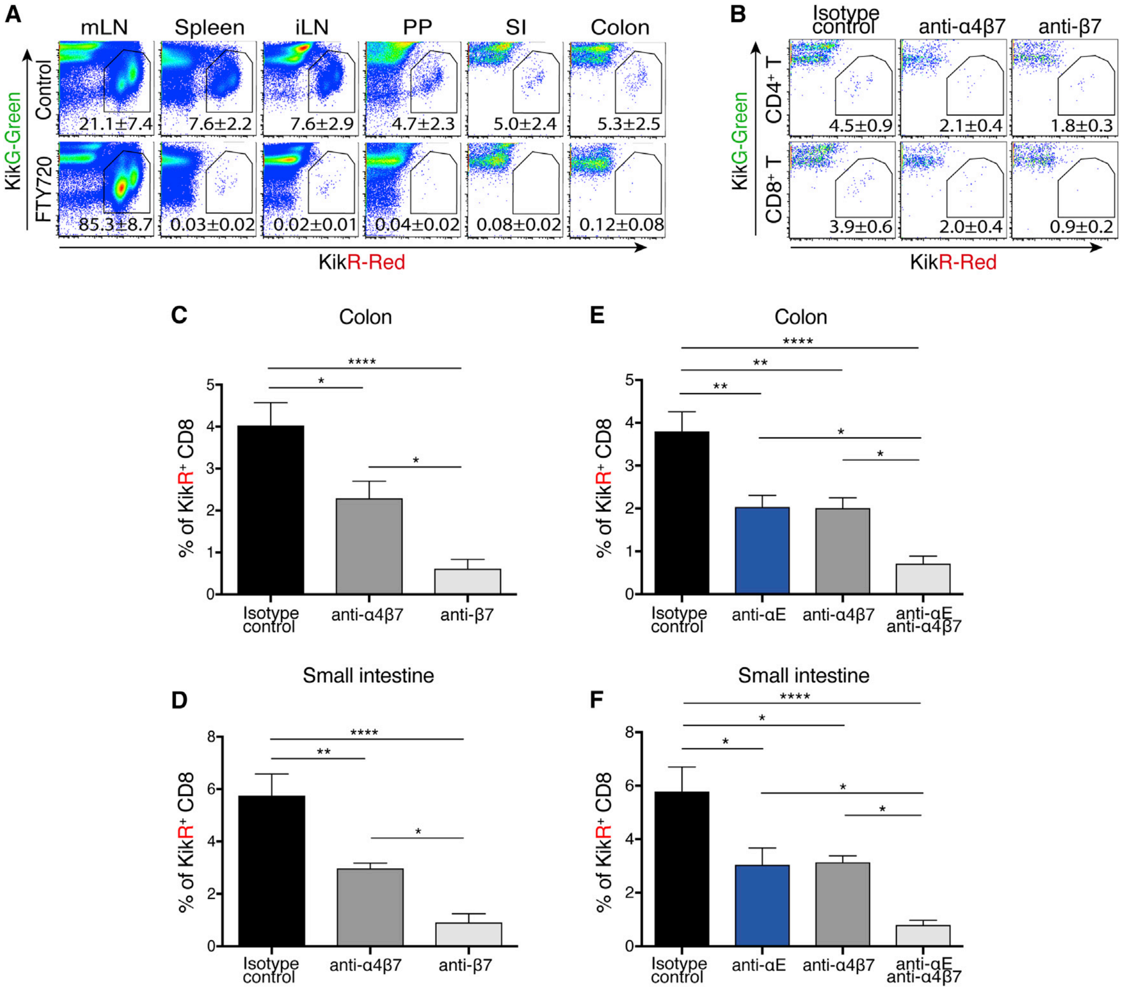

Figure 1. Blockade of β7 or α4β7 and αEβ7 reduces $CD8^+$ T cell accumulation in the small intestine and colon over α4β7 alone in the KikGR transgenic mouse model

(A) Lymphoid and gut tissues from control or FTY720-treated KikGR transgenic mice were analyzed 16 h after mLN photoconversion. Gated $CD45^+TCR\beta^+$ cells photoconverted in the mLN can be identified by their expression of KikR red fluorescent protein.

(B–D) Anti-integrin or isotype control antibodies were administered to KikGR transgenic mice before surgical photoconversion of mLN. Colonic $CD4^+$ (top) and $CD8^+$ (bottom) T cell accumulation was reduced by anti-integrin antibody treatment. Data are from a representative sample of six to eight mice analyzed (B). Reduction in $KikR^+$ $CD45^+TCR\beta^+CD8^+$ T cells from colon (C) and small intestine (D) after anti-β7 treatment in comparison with anti-α4β7 treatment.

(E and F) Isotype control, anti-αE, and/or anti-α4β7 integrin antibodies were administered to KikGR mice before surgical photoconversion of mLN. The percentage of photoconverted $KikR^+$ cells among $CD45^+TCR\beta^+CD8^+$ T cells in the colon (E) and small intestine (F) are shown.

Bar graph shows means ± SEM. Data are from six to eight animals combined from two to three independent experiments. *$p < 0.05$, **$p < 0.01$, ***$p < 0.001$ by one-way ANOVA with Tukey's multiple-comparison post-test.

were used to enable live cell imaging. OT-1-tdTomato T cells localized to the basement membrane in close proximity to basolateral surfaces of epithelial cells, with extended cell bodies suggesting activation (Figure 3B; Video S1). Upon blockade of αEβ7 or E-cadherin, OT-1-tdTomato T cells preferentially localize to the LP with a few T cells observed in the epithelium (Figure 3B).

To follow T cell-epithelial cell interactions over time, OT-1-tdTomato $CD8^+$ T cells were transferred into E-cadherin/cyan fluorescent protein (CFP) reporter mice, and activated T cells were tracked from the luminal side of the small intestine using two-photon intravital imaging (Figure 3C). As previously reported,[30,32] activated T cells migrated toward subepithelial regions and exhibited dynamic interactions with epithelial cells during movement across the basement membrane (Figure 3C; Video S2). Upon αEβ7 blockade, activated T cells were observed to migrate across the basement membrane to reach the subepithelial regions less frequently (Figure 3C; Video S3). Quantification of individual T cells showed a statistically significant increase in maximum track speed (Figure 3D) and track length (Figure 3E) after αEβ7 blockade in comparison with the control. Because the blockade of αEβ7 led to increased T cell motility and reduced T cell-epithelial cell contact, our findings are consistent with a critical role for αEβ7 and E-cadherin in

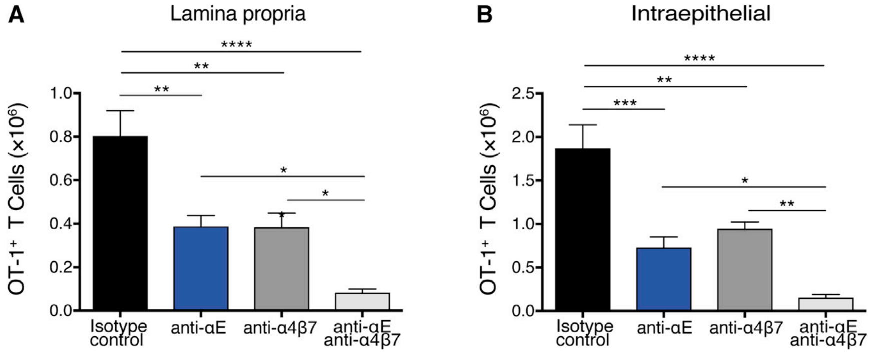

Figure 2. Reduction in both lamina propria and intraepithelial antigen-specific CD8$^+$ T cells with blockade of α4β7 and αEβ7 is increased over α4β7 blockade alone

KikG$^+$ green fluorescent ovalbumin-specific OT-1 TCR transgenic CD8$^+$ T cells were adoptively transferred into WT recipients that were then orally challenged with cholera toxin:OVA the following day. Three days after immunization, recipient mice were treated with isotype or anti-integrin antibodies 16 h before isolation of cells from the small intestine. The number of KikG$^+$ OT-1 cells in the LP (A) and intraepithelial (B) compartments after antibody treatment are shown. Bar graph shows means ± SEM of six to eight mice combined from two independent experiments. *p < 0.05, **p < 0.01, ***p < 0.001 by one-way ANOVA with Tukey's multiple-comparison post-test.

mediating tissue retention through sustained interactions between activated T cells and the epithelium.

Blockade of αEβ7 increases T cell migration from the gut to the mLN

Lymphatic vessels serve as conduits for lymphocyte movement out of the gut and into the mLN.[33,34] The decrease in tissue retention after αEβ7 blockade may, therefore, directly affect T cell egress through lymphatics. To test that hypothesis, activated KikGR$^+$OT-1$^+$ cells were photo-stamped within the small intestine to enable lymphocyte tracking, and both KikR$^+$OT-1 cells remaining in the small intestine and KikR$^+$OT-1 cells in the mLN were evaluated 16 h later (Figure S2A). αEβ7 or E-cadherin blockade led to an ~50% reduction in KikR$^+$OT-1 T cells in the small intestine (Figures 4A and S2B) and increased KikR$^+$ OT-1 cells in the MLN by 3-fold (Figures 4B and S2C). Treatment with anti-α4β7 antibody did not alter KikR$^+$ OT-1 cells in the small intestine or mLN in comparison with the control, but the total number of OT-1$^+$ cells was reduced, making the percentage similar to the FTY720 treatment (Figures 4A, 4B, S2B, and S2C).

T cell migration through the lymphatic vessels to the draining LNs is dependent on S1PR1.[11,33] Treatment with FTY720, a S1PR1 agonist, increased T cells in the small intestine and reduced T cells in the mLN in comparison with the control (Figures 4A, 4B, S2B, and S2C). Consistent with an effect on lymphocyte egress from the mucosa, photo-stamped T cells were rare in intestinal lymphatics of FTY720-treated mice. In contrast, αEβ7 blockade increased T cell frequency within the lymphatic vessels (Figures 4C and 4D). Taken together, these findings support a critical role for αEβ7, but not α4β7, in T cell retention within gut tissues, and the blockade increases subsequent egress of activated effector T cells from the mucosa to the draining LNs.

αEβ7 expression is restricted to cytotoxic CD8$^+$ IELs and proinflammatory CD4 T cell subsets in human colonic tissue

Studies in a photoconvertible mouse model demonstrated additive roles for α4β7 and αEβ7 integrins in T cell migration and retention, suggesting targeting those complementary mechanisms with anti-integrin therapies may be important in IBD. Analysis of αE-expressing pooled IEL and LP immune cells in healthy and IBD human colonic biopsies showed that, similar to the mouse, most CD8$^+$ T cells express αEβ7 (~80%), whereas only ~10% of the CD4$^+$ T population are αEβ7$^+$ (Figures 5A and S3A). αE$^+$CD4$^+$ T cells produced higher levels of inflammatory cytokines, including interleukin 17 (IL-17) and interferon γ (IFN-γ) as compared with αE$^-$CD4$^+$ T cells (Figures 5B and S3B). Human colonic regulatory T cells (CD4$^+$CD25^{high}CD127^{low-}Foxp3$^+$) have low to absent αEβ7 expression as compared with non-regulatory T cells, in contrast to the proinflammatory phenotype of αE$^+$CD4$^+$ T cells (Figures 5C and S3C). Comparison of human and mouse T cells, using the *Helicobacter hepaticus* anti-IL10R model of colitis,[35] suggested similarities in αE$^+$CD8$^+$ T cells (Figures 5H, S3D, and S3G), whereas the proinflammatory state of αE$^+$CD4$^+$ human T cells is directly opposite that of murine αE$^+$CD4$^+$ T cells, which have a regulatory T cell phenotype and produce significantly less inflammatory cytokines (Figures S3E and S3F).

Single-cell sequencing of biopsies containing both IELs and lamina propria lymphocytes (LPLs) revealed cellular heterogeneity of human colonic T cells with five main lymphocyte sub-clusters: CD8$^+$ IELs, LP CD8$^+$ TCRαβ$^+$, CD4$^+$ effector/memory T cells, CD4$^+$ regulatory T cells, and ILC3 cells (Figures 5D, S4A, and S4B; Data S1). The αE transcript is most abundant in CD8$^+$ IELs (Figure 5E), consistent with surface protein expression (Figure 5A), and cytotoxic genes, including granzyme A/B (GZMA and GZMB), perforin, and natural killer (NK) receptors (KLRD1 and NKG7), are also expressed (Figure 5E). Further sub-clustering of CD8$^+$ cytotoxic IELs identified four major subsets: CD8$^+$TCRγδ$^+$, CD8$^+$TCRαβ$^+$, CD8$^+$TCRαβ$^+$IFN-γ$^+$, and CD8$^+$TCRαβ$^+$TIGIT$^+$CD96$^+$ IELs (Figure S4C). Despite their distinct TCR usage, cytokine profiles, and inhibitory receptor expression, all four CD8$^+$ IEL subpopulations have similarly high expression of αE and cytotoxic genes, including GZMA and GZMB, perforin (PRF1), and NK receptors (KLRD1 and NKG7) (Figure S4D). Deep sequencing of sorted αE$^+$ or αE$^-$ CD8$^+$ T cells (Figure 5F) demonstrated αE$^+$CD8$^+$ IELs expressed even higher levels of cytotoxic genes compared with αE$^-$ CD8$^+$ IELs (Figure 5G). Flow cytometry confirmed those findings, with αE$^+$CD8$^+$ T cells exhibiting higher level of GZMA and

CellPress
OPEN ACCESS

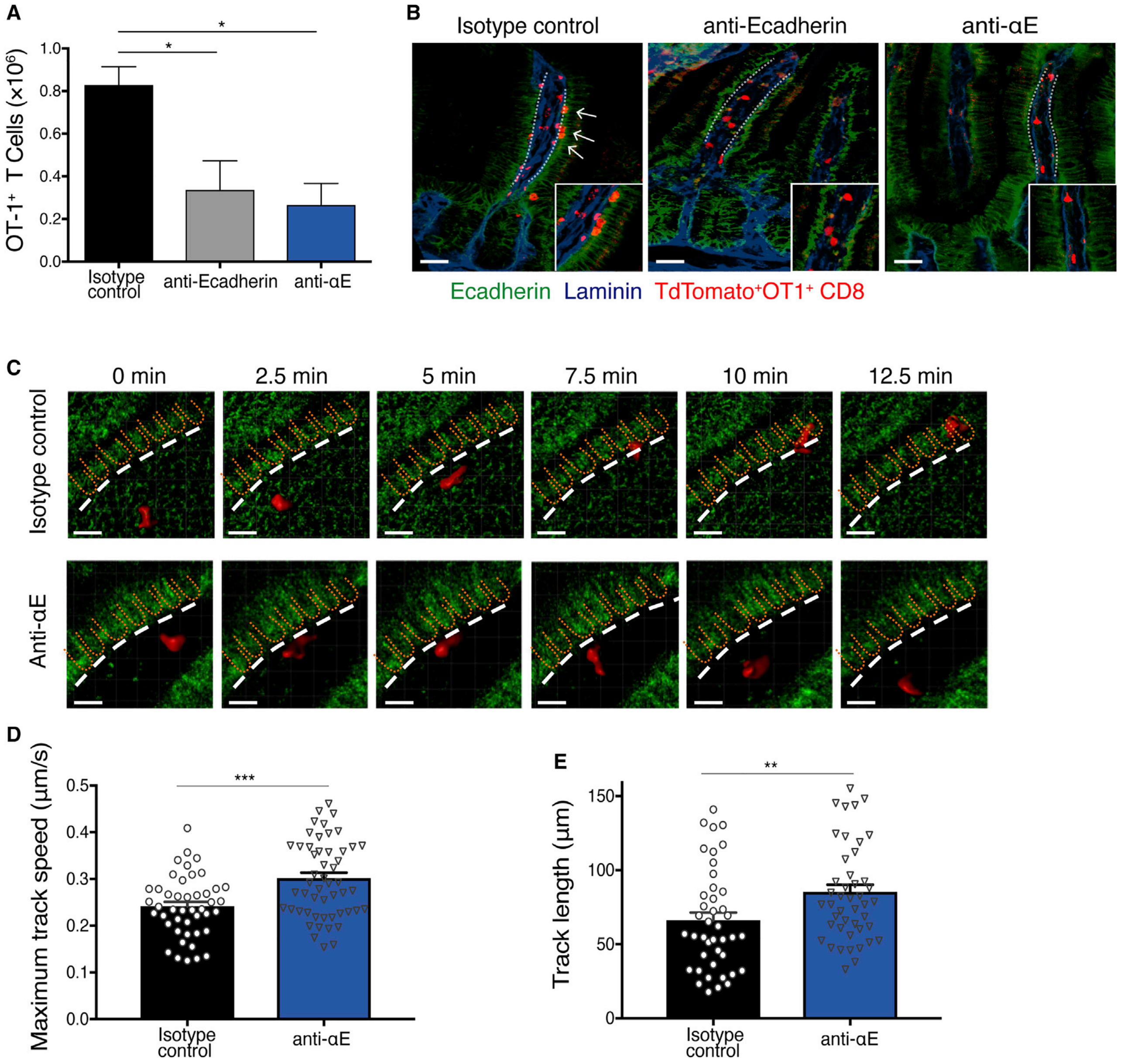

Figure 3. αEβ7 or E-cadherin blockade inhibits T cell interactions with intestinal epithelium

Adoptive transfer of tdTomato$^+$ OT-1 cells into WT or E-cadherin-CFP recipients was followed by oral challenge with cholera toxin:OVA the following day. Three days after immunization, recipient mice were treated with isotype or anti-αE or anti-E-cadherin antibodies before analysis.

(A) OT-1 cell number in the small intestine lamina propria. Bar graph shows means ± SEM of six to eight mice combined from two independent experiments.

(B) Confocal microscopy image of intestinal tissue sections stained with anti-E-cadherin (green) and anti-laminin (blue). White arrows point to tdTomato (red) OT-1 T cells in close proximity to E-cadherin on basolateral epithelium. Scale bar: 100 μm. Data are representative of three independent experiments.

(C) Representative movie of tdTomato (red) OT-1 T cells interacting with intestinal epithelium (green) in control or anti-αE-treated mice. The basement membrane is indicated by a dashed white line. Scale bar: 20 μm.

(D and E) Maximal track speed (D) and track length (E) were quantified for individual cells. Bar graph shows means ± SEM, and each dot represents an individual cell migration event. Data are representative of five independent experiments. One-way ANOVA with Dunnett's post-test or unpaired Student's t test was used to calculate statistical significance. *$p < 0.05$, **$p < 0.01$, ***$p < 0.001$.

GZMB, PRF1, NK receptors, and other tissue-resident T cell markers, such as CD49a (Figure 5H).[36] Taken together, our data indicate that αE expression by CD8$^+$ cytotoxic IELs is associated with expression of cytotoxic genes, IFN-γ, and chemokines CCL3–5.

Blockade of β7 integrin by etrolizumab reduces cytotoxic CD8$^+$ IEL gene expression in intestinal biopsies

Our mechanistic studies suggest β7 blockade may alter CD8$^+$ T cell subset accumulation in intestinal tissue in patients with IBD. We tested the effect of β7 blockade in patients with CD

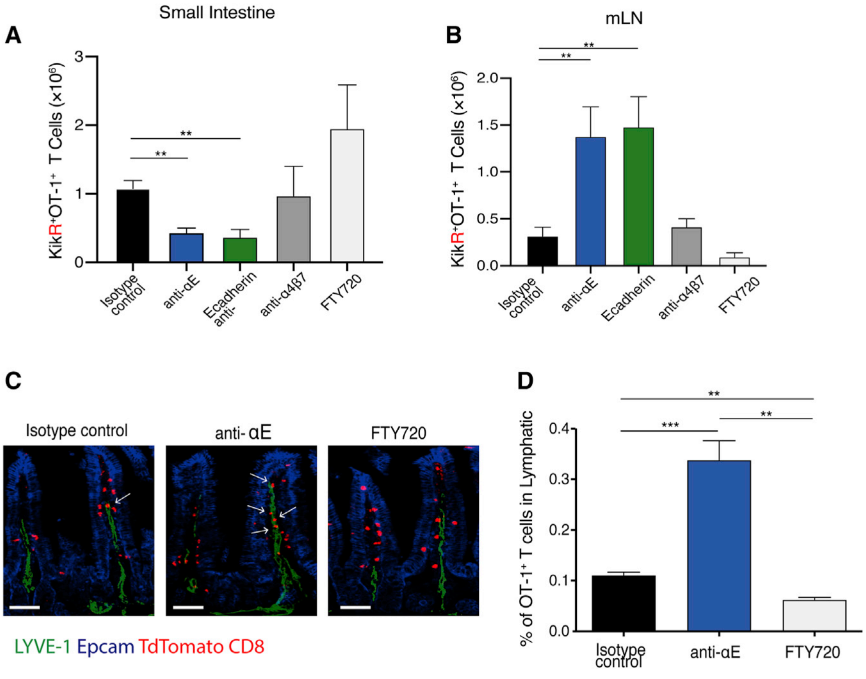

Figure 4. αE inhibition increases intestinal T cell egress from small intestine into mLN

(A and B) KikGR+ OT-1 cells were adoptively transferred into WT mice that were then orally challenged with cholera toxin:OVA. Three days after immunization, mice were treated with isotype or anti-αE integrin antibodies or FTY720, followed by a surgical photoconversion of the small intestine. Cells in the small intestine and mLN were evaluated 16 h after photoconversion by flow cytometry. Number of KikR+ OT-1 cells in small intestine (A) and mLN (B). Bar graphs show means ± (SEM) of six to eight animals combined from two to three independent experiments.

(C and D) tdTomato+ OT-1 cells were adoptively transferred into WT mice before oral challenge and antibody treatment as in (A) and (B). (C) Representative confocal microscopy image of intestinal tissue sections stained with anti-LYVE-1 (green) and anti-Epcam-1 (blue). White arrows show tdTomato (red) OT-1 T cells within lymphatic vessels. Scale bar: 100 μm. (D) The small intestine was sectioned, and tdTomato OT-1 T cells were counted in ~100 villus per mouse to determine the frequency of OT-1 cells within lymphatic vessels. Bar graph shows means ± SEM of three animals. Data are representative of three independent experiments. One-way ANOVA with Tukey's multiple-comparison post-test was used to calculate statistical significance. $*p < 0.05$, $**p < 0.01$, $***p < 0.001$, $****p < 0.0001$

who were enrolled in a phase III study of etrolizumab.[24] Colonic or ileal biopsies were taken for RNA-seq before randomization to etrolizumab or placebo treatment arms and at 14 weeks after treatment. Etrolizumab-treated patients showed significant decreases in integrin gene expression with reductions in β7 integrin (*ITGB7*) and α4 integrin (*ITGA4*) (Figure 6A) in both the ileum and the colon. Although decreased expression of αE integrin (*ITGAE*) was observed after etrolizumab treatment, the difference was not statistically significant relative to placebo (Figures 6A and S5A).

Significant post-treatment changes in gene expression were observed in etrolizumab-treated patients but not in placebo-treated controls (Figures 6B and 6C). Comparison of baseline gene expression between areas with active disease and areas without active disease showed an inverse correlation between genes altered by etrolizumab treatment and genes associated with endoscopic activity (Figures 6D and 6E), suggesting a treatment-specific reduction in active inflammation by etrolizumab. The genes most downregulated by etrolizumab include genes associated with B cells (IGHG1, PAX5, and CD79B), T cells (CD8A, CD5, and CD28), and inflammatory cytokines (IFN-γ, IL-23, and TNF) (Figure 6B).

Because CD can affect the entire gastrointestinal tract, subanalyses by anatomic region were performed. Gene expression changes between active and inactive disease were similar in both the ileum and colon Figure S5 (Figures S6A and S6B), and inflammatory gene expression was also similarly reduced in both locations after etrolizumab treatment Figures S5 (Figure S6C). These data demonstrate that inflammation-associated genes are downregulated by etrolizumab treatment in the colon and ileum. No correlation was observed in placebo-treated patients (Figure 6E).

To identify the T cell subsets affected by etrolizumab treatment, gene modules specific for each type of T cell were generated using our single-cell RNA-seq data (Figure 5D; Data S1). Signature scores were calculated for each of the gene modules in the bulk RNA-seq data from the CD cohort. CD8+ cytotoxic IEL and CD8+ LP T cell signature genes decreased in etrolizumab-treated patients in comparison with those treated with placebo (Figures 6F, S5B, and S7). Other T cell subsets also decreased from baseline in etrolizumab-treated patients, but there was no statistically significant difference between the etrolizumab and the placebo arms (Figure 6F). CD8+ cytotoxic IEL-specific genes, such as CD8A, NKG7, and CCL5, were strongly downregulated after etrolizumab treatment (Figures 6G and S5C).

Vedolizumab, which blocks α4β7 integrin, has been suggested to inhibit intestinal T cell trafficking, yet a recent study analyzing pre- and post-treatment colonic biopsies did not find any reduction in CD4+ and CD8+ T cells at 14 weeks.[37] A study in a small cohort of HIV-infected patients with IBD showed an increase in total T cells in the terminal ileum, with no change in CD4/CD8 ratio, after vedolizumab treatment.[38] To assess the effect of vedolizumab on cytotoxic IELs, we analyzed publicly available biopsy microarray data from patients with UC before and 12 weeks after treatment with vedolizumab.[39] Similar to Zeissig et al.[37], we did not observe a significant reduction of CD8+ cytotoxic IELs or CD8+ LP T cell-associated genes (Figure S7). Taken together with the reduction in CD8+ cytotoxic IELs and CD8+ LP T cell-associated genes after etrolizumab treatment, these data suggest that blockade of both α4β7 and

CellPress OPEN ACCESS

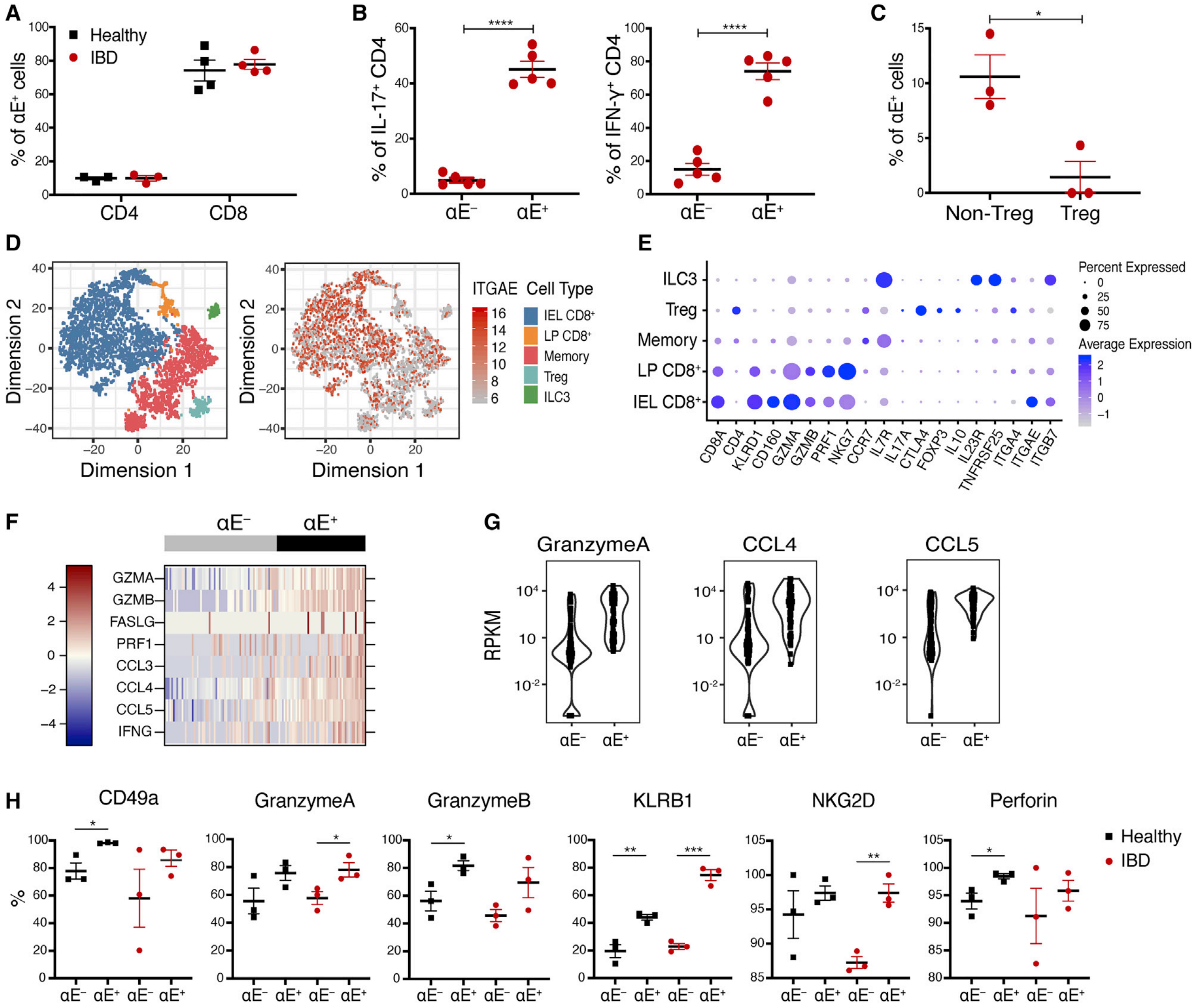

Figure 5. αEβ7 is highly expressed on human colonic CD8$^+$ T cells and proinflammatory CD4$^+$ T cells

(A) Percentage of αE$^+$ cells among CD4$^+$ and CD8$^+$ T cells from healthy or IBD colonic biopsies.

(B and C) Colonic αE$^+$ CD4$^+$ T cell and αE$^-$CD4$^+$ T cell production of IL-17 and IFN-γ (B) and percentage of αE$^+$ cells (C) among CD4$^+$Foxp3$^+$CD25$^+$CD127^{low} cells (Treg) and CD4$^+$ T cells. Each dot represents an individual patient sample, and data are shown as means ± SEM.

(D) Single-cell RNA-seq data from colonic biopsies from healthy subjects are shown as t-stochastic neighborhood embedding (t-SNE) of CD45$^+$ cells colored by cell subsets (left). Right, expression of αE in the corresponding cell population.

(E) T cell subsets and their expression of indicated genes. Dot size indicates the percentage of gene expression, whereas dot color signifies the mean expression level of selected marker genes across cell subsets.

(F and G) Deep sequencing of sorted αE$^+$CD8$^+$TCRβ$^+$ cells and αE$^-$CD8$^+$TCRβ$^+$ cells from a diverticulitis (non-IBD) resection sample at single-cell resolution. (F) Heat plot of expression of selected genes. (G) Reads per kilobase of transcript per million mapped reads (RPKM) of granzyme A (left), CCL4 (middle), and CCL5 (right) in αE$^+$ and αE$^-$ populations. $p < 1.0 \times 10^{-8}$ for all three genes.

(H) Quantification of selected markers in colonic αE$^+$ and αE$^-$ CD8$^+$ T cells by flow cytometry. *$p < 0.05$, **$p < 0.01$, ***$p < 0.001$, ****$p < 0.0001$ by unpaired Student's t test.

Human samples are from Stanford (A and H), UCSF (C–E), or the Mayo clinic (B, F, and G). See also Table S1.

αEβ7 is required to achieve a reduction in genes associated with CD8$^+$ cytotoxic IELs and CD8$^+$ LP T cells.

DISCUSSION

IBD is a chronic relapsing-remitting intestinal inflammatory disorder associated with an increase in activated lymphocytes in the intestinal mucosa.[13] Etrolizumab, an anti-β7 integrin monoclonal antibody, is being tested for efficacy in CD in phase III (ClinicalTrials.gov: NCT02394028). Previous reports using humanized mouse models have shown that α4β7 is critical for homing of T cells to the colon[40] and that blockade of αEβ7 can further decrease accumulation of CD8$^+$ T cells.[41] Here, we applied a surgical photoconversion system to quantify T cell homing and

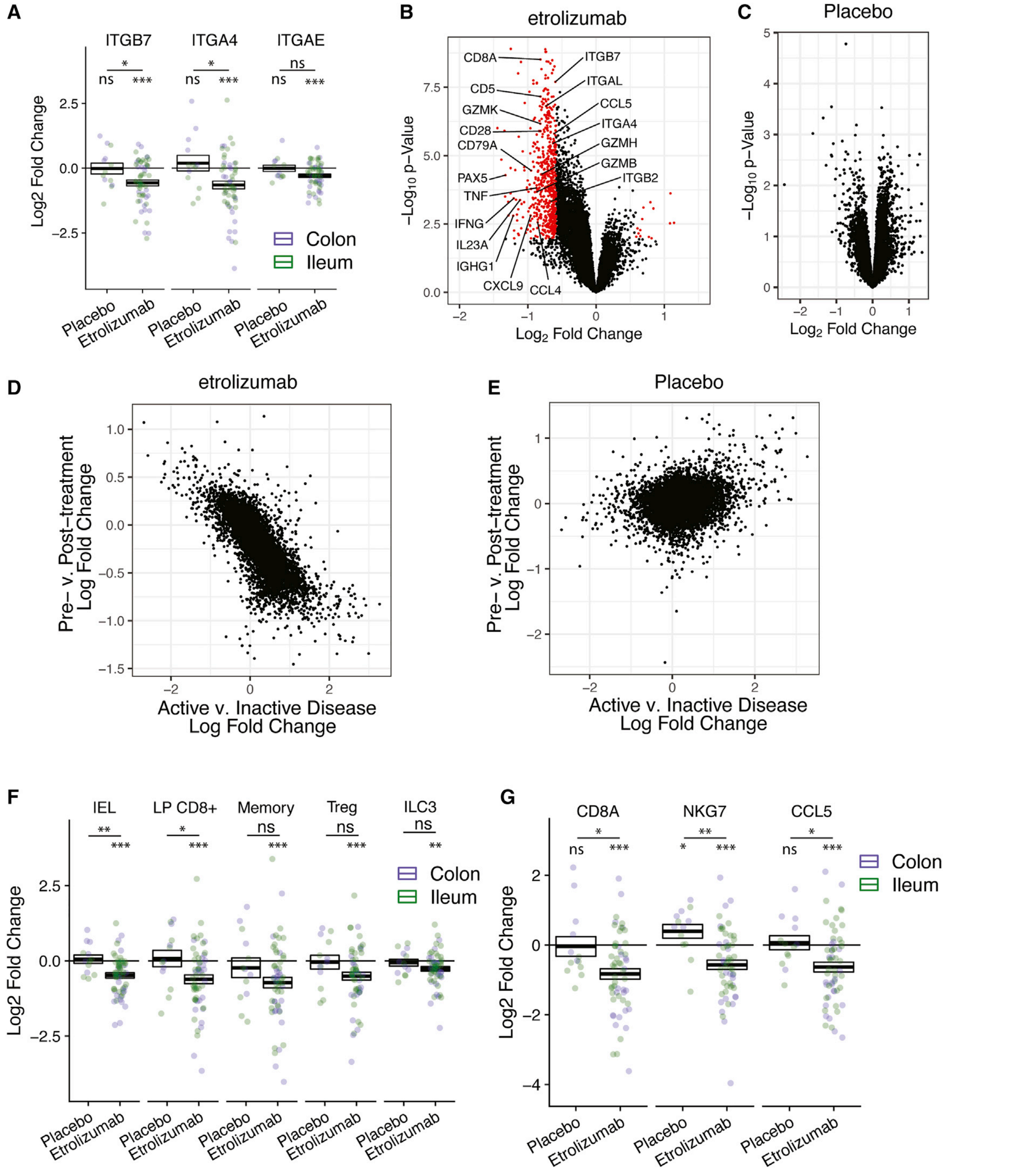

Figure 6. Etrolizumab treatment significantly reduces expression of genes associated with CD8$^+$ cytotoxic IELs

Ileal or colonic biopsies were taken before treatment and at 14 weeks after treatment in a randomized placebo-controlled trial of etrolizumab (anti-β7 integrin) in patients with moderately to severely active CD.

(A) Fold change of integrin genes at 14 weeks after etrolizumab or placebo treatment. Each dot is an individual patient sample pair. Points are colored by sampling location (blue, colon; green, ileum). Boxes show the standard error, with the middle bar showing the group mean value.

(B and C) Volcano plots showing the $\log_2$-fold change and p value of each gene, comparing baseline to week-14 samples from etrolizumab-treated patients (B) or placebo-treated patients (C). Each gene is represented by a point, with genes shown in red undergoing a >1.5-fold change at a false-discovery rate (FDR) < 0.05.

(D and E) Scatterplots showing the correlation of the $\log_2$-fold changes of individual genes in endoscopically active, versus inactive, disease and the $\log_2$-fold changes observed between screening and week-14 samples from (D) etrolizumab-treated patients or (E) placebo-treated patients. Each point represents an individual gene.

(legend continued on next page)

retention and found that a combined blockade of α4β7 and αEβ7 is more effective in reducing $CD8^+$ T cell accumulation in gut tissue than is blocking either integrin alone in an antigen-specific mouse model. Further, αEβ7 and E-cadherin mediate interactions of activated $CD8^+$ T cells with the basolateral epithelium and prolong tissue retention time through inhibiting intestinal tissue egress. Our findings confirm that blockade of α4β7 and αEβ7 integrins inhibit T cell intestinal accumulation and extend their role to tissue entry and retention in a respective stepwise manner to inhibit T cell accumulation in the gut mucosa in a mouse model. Single-cell RNA-seq and flow cytometry of human colonic T cells show that most αE^+ T cells are pro-inflammatory. Intestinal biopsy data from a placebo-controlled study of patients with CD[26] demonstrate etrolizumab-specific reduction in genes associated with gut $CD8^+$ cytotoxic IELs, along with a broad decrease of genes associated with active inflammation, consistent with a role for α4β7 and αEβ7 integrins in CD8 T cell accumulation in the gut mucosa.

Gastrointestinal immune priming and tissue trafficking is achieved through the induction of integrin and chemokine receptors that mediate gut homing of T cells activated within intestinal-draining lymphoid organs. Integrin-dependent gut trafficking is not all encompassing. T cells can still migrate to the gut during inflammation in the absence of α4β7 expression,[42] and anti-α4β7 blockade only partially reduces T cell trafficking to the gut (Figure 1). Integrins α4β1 and αLβ2 may also mediate intestinal homing, a mechanism of particular importance in the ileum.[43] Expression of addressins VCAM-1 and ICAM-1 on intestinal vascular endothelium can mediate adhesion and may be upregulated under inflammatory conditions,[44] although reports in IBD are mixed.[45,46] $\alpha E\beta 7^+$ dendritic cells (DCs) have been shown to have reduced induction of regulatory T cells and increased induction of pro-inflammatory T cell responses in patients with IBD.[47] Once T cells upregulate αE expression, their ability to interact with the epithelium through direct binding between αEβ7 and E-cadherin on the basolateral side of the intestinal epithelium is increased, and previous studies have shown αE-dependent accumulation of $CD8^+$ and Th9 T cells in a humanized mouse model.[41] Our data are consistent with a role for αEβ7 and E-cadherin interactions in T cell migration across the intestinal basement membrane into the intraepithelial space to enable tissue retention, which could be through direct or indirect effects. In the mechanistic murine studies presented here, inhibition of both α4β7-dependent cell entry and αEβ7-dependent retention leads to a greater reduction of T cells in the gut over inhibition of either pathway alone.

There are important differences in αE expression between human and mouse $CD4^+$ T cell lineages, which we have directly assessed here. Human intestinal regulatory T cells have low to absent expression of αEβ7, whereas murine regulatory T cells express a high level of αEβ7 (Figures 5 and S3E). In addition, human αE^+CD4^+ T cells are more polyfunctional with a higher proportion of cells that produce proinflammatory cytokines, including IL-17 and IFN-γ[48] (Figure 5). In both human and mouse, most gut $CD8^+$ T cells express high levels of αEβ7; however, human $CD8^+$ IELs express much higher levels of cytotoxicity-associated genes (Figures 5 and S3G). These differences highlight the importance of cross-validation of observations between pre-clinical and clinical studies to inform our mechanistic understanding of therapies in IBD. It is also critical to continue to validate observations, such as the relative roles of α4β7 and αEβ7 in integrin-mediated homing and retention made in model systems directly in human clinical studies wherever possible, with particular attention to new methodologies that can increase our mechanistic understanding.

Although anti-integrin therapies have been suggested to block trafficking of lymphocytes into the intestinal mucosa, data from patients treated with anti-integrins has been mixed. One study showed a reduction in intestinal $CD4^+$ T cells in patients with CD treated with natalizumab (anti-α4) using immunohistochemistry.[49] More recently, patients with UC and those with CD underwent a cell-labeling and imaging approach before and after vedolizumab (anti-α4β7) treatment, and no change in T cell migration to the intestinal mucosa was observed.[37] In the same study, no change in T cell frequency was observed when evaluated by immune cell phenotyping by flow cytometry after 14 weeks of vedolizumab treatment.[37] Vedolizumab treatment in HIV^+ patients with IBD resulted in an increase in T cells in the ileum as well as a striking decrease in the size of lymphoid aggregates. Although HIV-1 tropism for mucosal homing cells complicates the interpretation, the decrease in lymphoid aggregate size, which may be more dependent on α4β7, instead of mucosal T cells, is notable.[38]

In the phase III cohort of patients with CD assessed here, as well as in a previous phase II study in patients with UC,[23] etrolizumab reduced expression of T cell genes in intestinal mucosa after 14 and 10 weeks, respectively. We applied the same methodology to a second vedolizumab study in patients with UC[39] (Figure S7) and confirmed that no change of T cell-associated gene sets was observed 12 weeks after treatment. Possible explanations for this negative finding may be the reduced dynamic range for measuring gene-expression changes inherent to microarrays in comparison with RNA-seq[50] or that blockade of α4β7-dependent cell entry by vedolizumab may take longer than 12 weeks to have a noticeable effect on colonic T cells. However, our analysis showing T cell genes do not decrease after vedolizumab treatment is consistent with the Zeissig et al.[37] study and, together with our murine data, suggests the reduction of cytotoxic IEL genes by etrolizumab may be attributable to the additional blockade of αEβ7-dependent tissue retention, which may contribute to greater or faster inhibition of inflammation during induction therapy with etrolizumab compared with vedolizumab. Future clinical studies with head-to-head comparison of etrolizumab and vedolizumab will be required to fully evaluate the differences between these therapeutic approaches.

The reduction in $CD8^+$ cytotoxic IELs observed only in etrolizumab-treated patients suggests a potential effect of etrolizumab on the dysregulated mucosal immune response in IBD. $CD8^+$

(F and G) Fold change of expression of the signature gene sets of indicated T cell subtype gene sets (F) and the cytotoxic IEL-specific genes (G) at week 14 in patients treated with etrolizumab or placebo. Each dot represents an individual patient sample pair. Points are colored by sampling location (blue, colon; green, ileum). Boxes show the standard error, with the middle bar showing the group mean value. *$p < 0.05$, **$p < 0.01$, ***$p < 0.001$.

T cells have been associated with prognostic outcomes in IBD,[51] and the role of IELs, which are predominantly CD8$^+$, in inflammation is beginning to be better appreciated. CD8$^+$ cytotoxic IELs are closely associated with the intestinal epithelium and, under normal conditions, can quickly provide defense against pathogen dissemination[31,52] because of their resting expression of cytotoxic genes, which enables them to respond without proliferation.[53,54] Upon stimulation, rapid IFN-γ secretion by CD8$^+$ cytotoxic IELs augments T cell cytotoxicity against infected epithelial cells and increases barrier permeability.[55–58] Additionally, gut-resident CD8$^+$ T cells express high levels of NK receptors, such as NKG2D and KLRD1, which enable recognition of stressed epithelial cells.[59] Two recent studies of CD8$^+$ T cells from healthy volunteers and patients with UC using single-cell (sc)RNA-seq identified αEβ7$^+$ effector TNF-α$^+$IFN-γ$^+$CD8$^+$ T cells.[60,61] It has long been recognized that the frontline role of cytotoxic IELs is not without risks of aberrant activation.[62] and recent data show that chronic inflammation in celiac disease, a gastrointestinal disorder triggered by immune response to dietary gluten, induces a pathogenic phenotype in IELs characterized by increased IFN-γ production and expression of CCL4.[63] Whether similar effects of chronic inflammation on cytotoxic IEL populations occur in IBD will require further investigation but may soon yield to scRNA-seq techniques able to probe CD8$^+$ cellular heterogeneity.

In conclusion, we demonstrate that integrins α4β7 and αEβ7 have cooperative roles in CD8$^+$ T cell accumulation in the gut mucosa in a murine model of T cell trafficking. These data highlight an underappreciated role for αEβ7 in T cell retention at the epithelial barrier through T cell-epithelial interactions that inhibit S1PR1-dependent tissue egress and migration to mLN. Key differences between human and mouse αEβ7 expression on CD4$^+$ T cell lineages, particularly regulatory T cells, were identified. Finally, we observed a significant reduction in expression of gene sets associated with CD8$^+$ cytotoxic IELs after treatment with anti-β7 etrolizumab therapy in a large phase III study of patients with CD. Although recent data from phase III UC studies of etrolizumab showed mixed efficacy in top-line clinical efficacy,[64] clinical studies of etrolizumab in CD are on-going.

Limitations of the study

Recognizing that mouse models have limitations in their ability to recapitulate human biology is important to acknowledge. Here, we report data using mouse-surrogate antibodies to dissect migration to, and retention of, lymphocytes in the intestinal mucosa, which show a role for α4β7 and αEβ7 in T cell trafficking, suggesting that the therapeutic potential in IBD may be greater if both integrin heterodimers are blocked. Because of the technical challenges of photo-conversion, a key component of the experimental model, we focused on the small intestine because cell retention in the colon could not be extensively investigated. In addition, possible depletion effects of murine surrogate antibodies have not been rigorously tested and could be mitigated by use of Fcγ receptor (FcGR)-deficient mice as recipients in experiments. Patient data from a clinical study of etrolizumab, which blocks both α4β7 and αEβ7, provide correlative data on the effectiveness of β7 blockade in reducing intestinal CD8 T cell populations analogous to our observations in murine models. There are limitations in the interpretation of human data based on sample size numbers, particularly given the number of paired samples in subanalyses based on treatment group and anatomic location, which will require validation in on-going etrolizumab trials.

STAR★METHODS

Detailed methods are provided in the online version of this paper and include the following:

- KEY RESOURCES TABLE
- RESOURCE AVAILABILITY
  - Lead contact
  - Materials availability
  - Data and code availability
- EXPERIMENTAL MODEL AND SUBJECT DETAILS
  - Mice
  - Patient samples
  - Etrolizumab clinical study in CD
- METHOD DETAILS
  - Blocking antibodies for murine studies
  - Flow cytometry antibodies and staining
  - Murine surgical photoconversion models of T cell trafficking
  - T cell adoptive transfer and oral antigenic challenge
  - Isolation and quantification of murine IEL and LP lymphocytes
  - Intra-vital two-photon microscopy live imaging
  - Immunofluorescence and confocal microscopy
  - *Helicobacter hepaticus* anti-IL10R induced colitis model
  - Preparation of cells from patient tissue samples for flow cytometry
  - Preparation of tissue samples for RNA-seq
  - Single cell RNA-seq analysis
  - Bulk RNA-seq analyses
- QUANTIFICATION AND STATISTICAL ANALYSIS
- ADDITIONAL RESROUCES

SUPPLEMENTAL INFORMATION

Supplemental information can be found online at https://doi.org/10.1016/j.xcrm.2021.100381.

ACKNOWLEDGMENTS

We thank Amanda Tatro, Oliwia Zurek, Zhiyong Yang, and Andrew Chan for critical review and editorial suggestions for this manuscript. The art work in the graphic abstract was assisted by Apothecom. This research was supported and funded by Genentech, Inc., USA.

AUTHOR CONTRIBUTIONS

B.D. performed experiments and analyzed data, J.A.H. analyzed data; R.I., K.-H.S., and A.G. performed experiments; A.N. processed patient samples; J.E., L.D.O., A.S., and Z.M. analyzed data; J.G., A.H., M.D., M.S., and W.A.F. were responsible for enrollment and acquisition of patient samples; A.C., Z.S., A.H., Y.S.O., S.T., and J.M. designed and executed the clinical studies; B.D., J.A.H., M.E.K., and T.Y. designed experiments and wrote the manuscript; all authors were involved in manuscript editing and finalization.

CellPress
OPEN ACCESS

DECLARATION OF INTERESTS

All authors, except J.G., A.H., M.D., M.S., and W.A.F., are current or past employees of Genentech, a member of the Roche group, and may hold Roche stock or stock options.

Received: June 12, 2020
Revised: March 9, 2021
Accepted: July 22, 2021
Published: August 17, 2021

REFERENCES

1. Zabel, B.A., Rott, A., and Butcher, E.C. (2015). Leukocyte chemoattractant receptors in human disease pathogenesis. Annu. Rev. Pathol. *10*, 51–81.
2. Johansson-Lindbom, B., Svensson, M., Wurbel, M.A., Malissen, B., Márquez, G., and Agace, W. (2003). Selective generation of gut tropic T cells in gut-associated lymphoid tissue (GALT): requirement for GALT dendritic cells and adjuvant. J. Exp. Med. *198*, 963–969.
3. Mora, J.R., Bono, M.R., Manjunath, N., Weninger, W., Cavanagh, L.L., Rosemblatt, M., and Von Andrian, U.H. (2003). Selective imprinting of gut-homing T cells by Peyer's patch dendritic cells. Nature *424*, 88–93.
4. Berlin, C., Berg, E.L., Briskin, M.J., Andrew, D.P., Kilshaw, P.J., Holzmann, B., Weissman, I.L., Hamann, A., and Butcher, E.C. (1993). Alpha 4 beta 7 integrin mediates lymphocyte binding to the mucosal vascular addressin MAdCAM-1. Cell *74*, 185–195.
5. El-Asady, R., Yuan, R., Liu, K., Wang, D., Gress, R.E., Lucas, P.J., Drachenberg, C.B., and Hadley, G.A. (2005). TGF-β-dependent CD103 expression by CD8$^+$ T cells promotes selective destruction of the host intestinal epithelium during graft-versus-host disease. J. Exp. Med. *201*, 1647–1657.
6. Hadley, G.A., Rostapshova, E.A., Gomolka, D.M., Taylor, B.M., Bartlett, S.T., Drachenberg, C.I., and Weir, M.R. (1999). Regulation of the epithelial cell-specific integrin, CD103, by human CD8$^+$ cytolytic T lymphocytes. Transplantation *67*, 1418–1425.
7. Cepek, K.L., Shaw, S.K., Parker, C.M., Russell, G.J., Morrow, J.S., Rimm, D.L., and Brenner, M.B. (1994). Adhesion between epithelial cells and T lymphocytes mediated by E-cadherin and the αEβ7 integrin. Nature *372*, 190–193.
8. Doğan, A., Wang, Z.D., and Spencer, J. (1995). E-cadherin expression in intestinal epithelium. J. Clin. Pathol. *48*, 143–146.
9. Higgins, J.M., Mandlebrot, D.A., Shaw, S.K., Russell, G.J., Murphy, E.A., Chen, Y.T., Nelson, W.J., Parker, C.M., and Brenner, M.B. (1998). Direct and regulated interaction of integrin alphaEbeta7 with E-cadherin. J. Cell Biol. *140*, 197–210.
10. Jaensson, E., Uronen-Hansson, H., Pabst, O., Eksteen, B., Tian, J., Coombes, J.L., Berg, P.L., Davidsson, T., Powrie, F., Johansson-Lindbom, B., and Agace, W.W. (2008). Small intestinal CD103$^+$ dendritic cells display unique functional properties that are conserved between mice and humans. J. Exp. Med. *205*, 2139–2149.
11. Laidlaw, B.J., Gray, E.E., Zhang, Y., Ramírez-Valle, F., and Cyster, J.G. (2019). Sphingosine-1-phosphate receptor 2 restrains egress of γδ T cells from the skin. J. Exp. Med. *216*, 1487–1496.
12. Shiow, L.R., Rosen, D.B., Brdicková, N., Xu, Y., An, J., Lanier, L.L., Cyster, J.G., and Matloubian, M. (2006). CD69 acts downstream of interferon-α/β to inhibit S1P1 and lymphocyte egress from lymphoid organs. Nature *440*, 540–544.
13. Adams, D.H., and Eksteen, B. (2006). Aberrant homing of mucosal T cells and extra-intestinal manifestations of inflammatory bowel disease. Nat. Rev. Immunol. *6*, 244–251.
14. Lamb, C.A., O'Byrne, S., Keir, M.E., and Butcher, E.C. (2018). Gut-selective integrin-targeted therapies for inflammatory bowel disease. J. Crohn's Colitis *12* (*suppl_2*), S653–S668.
15. Neurath, M.F. (2019). Targeting immune cell circuits and trafficking in inflammatory bowel disease. Nat. Immunol. *20*, 970–979.
16. Ley, K., Rivera-Nieves, J., Sandborn, W.J., and Shattil, S. (2016). Integrin-based therapeutics: biological basis, clinical use and new drugs. Nat. Rev. Drug Discov. *15*, 173–183.
17. Ghosh, S., Goldin, E., Gordon, F.H., Malchow, H.A., Rask-Madsen, J., Rutgeerts, P., Vyhnálek, P., Zádorová, Z., Palmer, T., and Donoghue, S.; Natalizumab Pan-European Study Group (2003). Natalizumab for active Crohn's disease. N. Engl. J. Med. *348*, 24–32.
18. Feagan, B.G., Rutgeerts, P., Sands, B.E., Hanauer, S., Colombel, J.F., Sandborn, W.J., Van Assche, G., Axler, J., Kim, H.J., Danese, S., et al.; GEMINI 1 Study Group (2013). Vedolizumab as induction and maintenance therapy for ulcerative colitis. N. Engl. J. Med. *369*, 699–710.
19. Sandborn, W.J., Feagan, B.G., Rutgeerts, P., Hanauer, S., Colombel, J.F., Sands, B.E., Lukas, M., Fedorak, R.N., Lee, S., Bressler, B., et al.; GEMINI 2 Study Group (2013). Vedolizumab as induction and maintenance therapy for Crohn's disease. N. Engl. J. Med. *369*, 711–721.
20. Dotan, I., Allez, M., Danese, S., Keir, M., Tole, S., and McBride, J. (2020). The role of integrins in the pathogenesis of inflammatory bowel disease: approved and investigational anti-integrin therapies. Med. Res. Rev. *40*, 245–262.
21. Rutgeerts, P.J., Fedorak, R.N., Hommes, D.W., Sturm, A., Baumgart, D.C., Bressler, B., Schreiber, S., Mansfield, J.C., Williams, M., Tang, M., et al. (2013). A randomised phase I study of etrolizumab (rhuMAb β7) in moderate to severe ulcerative colitis. Gut *62*, 1122–1130.
22. Tang, M.T., Keir, M.E., Erickson, R., Stefanich, E.G., Fuh, F.K., Ramirez-Montagut, T., McBride, J.M., and Danilenko, D.M. (2018). Review article: nonclinical and clinical pharmacology, pharmacokinetics and pharmacodynamics of etrolizumab, an anti-β7 integrin therapy for inflammatory bowel disease. Aliment. Pharmacol. Ther. *47*, 1440–1452.
23. Vermeire, S., O'Byrne, S., Keir, M., Williams, M., Lu, T.T., Mansfield, J.C., Lamb, C.A., Feagan, B.G., Panes, J., Salas, A., et al. (2014). Etrolizumab as induction therapy for ulcerative colitis: a randomised, controlled, phase 2 trial. Lancet *384*, 309–318.
24. Sandborn, W.J., Vermeire, S., Tyrrell, H., Hassanali, A., Lacey, S., Tole, S., and Tatro, A.R.; Etrolizumab Global Steering Committee (2020). Etrolizumab for the treatment of ulcerative colitis and Crohn's disease: an overview of the phase 3 clinical program. Adv. Ther. *37*, 3417–3431.
25. Rivera-Nieves, J. (2015). Strategies that target leukocyte traffic in inflammatory bowel diseases: recent developments. Curr. Opin. Gastroenterol. *31*, 441–448.
26. Sandborn, W.J., Panes, J., Jones, J., Hassanali, A., Jacob, R., Sharafali, Z., Oh, Y.S., and Tole, S. (2017). LBO3: etrolizumab as induction therapy in moderate to serve Crohn's disease: results from BERGAMOT cohort 1. United European Gastroenterol. J. *5*, 1138–1150.
27. Nowotschin, S., and Hadjantonakis, A.K. (2009). Use of KikGR a photoconvertible green-to-red fluorescent protein for cell labeling and lineage analysis in ES cells and mouse embryos. BMC Dev. Biol. *9*, 49.
28. Karecla, P.I., Bowden, S.J., Green, S.J., and Kilshaw, P.J. (1995). Recognition of E-cadherin on epithelial cells by the mucosal T cell integrin αM290 β7 (αEβ7). Eur. J. Immunol. *25*, 852–856.
29. Wurbel, M.A., Malissen, M., Guy-Grand, D., Malissen, B., and Campbell, J.J. (2007). Impaired accumulation of antigen-specific CD8 lymphocytes in chemokine CCL25-deficient intestinal epithelium and lamina propria. J. Immunol. *178*, 7598–7606.
30. Edelblum, K.L., Shen, L., Weber, C.R., Marchiando, A.M., Clay, B.S., Wang, Y., Prinz, I., Malissen, B., Sperling, A.I., and Turner, J.R. (2012). Dynamic migration of γδ intraepithelial lymphocytes requires occludin. Proc. Natl. Acad. Sci. USA *109*, 7097–7102.
31. Hoytema van Konijnenburg, D.P., Reis, B.S., Pedicord, V.A., Farache, J., Victora, G.D., and Mucida, D. (2017). Intestinal epithelial and intraepithelial T Cell crosstalk mediates a dynamic response to infection. Cell *171*, 783–794.e13.

32. Sujino, T., London, M., Hoytema van Konijnenburg, D.P., Rendon, T., Buch, T., Silva, H.M., Lafaille, J.J., Reis, B.S., and Mucida, D. (2016). Tissue adaptation of regulatory and intraepithelial CD4+ T cells controls gut inflammation. Science *352*, 1581–1586.

33. Huang, Y., Mao, K., Chen, X., Sun, M.A., Kawabe, T., Li, W., Usher, N., Zhu, J., Urban, J.F., Jr., Paul, W.E., and Germain, R.N. (2018). S1P-dependent interorgan trafficking of group 2 innate lymphoid cells supports host defense. Science *359*, 114–119.

34. Morton, A.M., Sefik, E., Upadhyay, R., Weissleder, R., Benoist, C., and Mathis, D. (2014). Endoscopic photoconversion reveals unexpectedly broad leukocyte trafficking to and from the gut. Proc. Natl. Acad. Sci. USA *111*, 6696–6701.

35. Kullberg, M.C., Jankovic, D., Feng, C.G., Hue, S., Gorelick, P.L., McKenzie, B.S., Cua, D.J., Powrie, F., Cheever, A.W., Maloy, K.J., and Sher, A. (2006). IL-23 plays a key role in *Helicobacter hepaticus*-induced T cell-dependent colitis. J. Exp. Med. *203*, 2485–2494.

36. Cheuk, S., Schlums, H., Gallais Sérézal, I., Martini, E., Chiang, S.C., Marquardt, N., Gibbs, A., Detlofsson, E., Introini, A., Forkel, M., et al. (2017). CD49a expression defines tissue-resident CD8+ T cells poised for cytotoxic function in human skin. Immunity *46*, 287–300.

37. Zeissig, S., Rosati, E., Dowds, C.M., Aden, K., Bethge, J., Schulte, B., Pan, W.H., Mishra, N., Zuhayra, M., Marx, M., et al. (2019). Vedolizumab is associated with changes in innate rather than adaptive immunity in patients with inflammatory bowel disease. Gut *68*, 25–39.

38. Uzzan, M., Tokuyama, M., Rosenstein, A.K., Tomescu, C., SahBandar, I.N., Ko, H.M., Leyre, L., Chokola, A., Kaplan-Lewis, E., Rodriguez, G., et al. (2018). Anti-α4β7 therapy targets lymphoid aggregates in the gastrointestinal tract of HIV-1-infected individuals. Sci. Transl. Med. *10*, eaau4711.

39. Arijs, I., De Hertogh, G., Lemmens, B., Van Lommel, L., de Bruyn, M., Vanhove, W., Cleynen, I., Machiels, K., Ferrante, M., Schuit, F., et al. (2018). Effect of vedolizumab (anti-α4β7-integrin) therapy on histological healing and mucosal gene expression in patients with UC. Gut *67*, 43–52.

40. Fischer, A., Zundler, S., Atreya, R., Rath, T., Voskens, C., Hirschmann, S., López-Posadas, R., Watson, A., Becker, C., Schuler, G., et al. (2016). Differential effects of α4β7 and GPR15 on homing of effector and regulatory T cells from patients with UC to the inflamed gut *in vivo*. Gut *65*, 1642–1664.

41. Zundler, S., Schillinger, D., Fischer, A., Atreya, R., López-Posadas, R., Watson, A., Neufert, C., Atreya, I., and Neurath, M.F. (2017). Blockade of αEβ7 integrin suppresses accumulation of CD8+ and Th9 lymphocytes from patients with IBD in the inflamed gut *in vivo*. Gut *66*, 1936–1948.

42. Kuklin, N.A., Rott, L., Darling, J., Campbell, J.J., Franco, M., Feng, N., Müller, W., Wagner, N., Altman, J., Butcher, E.C., and Greenberg, H.B. (2000). $\alpha_4\beta_7$ independent pathway for CD8+ T cell-mediated intestinal immunity to rotavirus. J. Clin. Invest. *106*, 1541–1552.

43. Zundler, S., Fischer, A., Schillinger, D., Binder, M.T., Atreya, R., Rath, T., Lopez-Pósadas, R., Voskens, C.J., Watson, A., Atreya, I., et al. (2017). The α4β1 homing pathway is essential for ileal homing of Crohn's disease effector T cells *in vivo*. Inflamm. Bowel Dis. *23*, 379–391.

44. Collins, T., Read, M.A., Neish, A.S., Whitley, M.Z., Thanos, D., and Maniatis, T. (1995). Transcriptional regulation of endothelial cell adhesion molecules: NF-κB and cytokine-inducible enhancers. FASEB J. *9*, 899–909.

45. Koizumi, M., King, N., Lobb, R., Benjamin, C., and Podolsky, D.K. (1992). Expression of vascular adhesion molecules in inflammatory bowel disease. Gastroenterology *103*, 840–847.

46. Nakamura, S., Ohtani, H., Watanabe, Y., Fukushima, K., Matsumoto, T., Kitano, A., Kobayashi, K., and Nagura, H. (1993). In situ expression of the cell adhesion molecules in inflammatory bowel disease. evidence of immunologic activation of vascular endothelial cells. Lab. Invest. *69*, 77–85.

47. Matsuno, H., Kayama, H., Nishimura, J., Sekido, Y., Osawa, H., Barman, S., Ogino, T., Takahashi, H., Haraguchi, N., Hata, T., et al. (2017). CD103+ dendritic cell function is altered in the colons of patients with ulcerative colitis. Inflamm. Bowel Dis. *23*, 1524–1534.

48. Lamb, C.A., Mansfield, J.C., Tew, G.W., Gibbons, D., Long, A.K., Irving, P., Diehl, L., Eastham-Anderson, J., Price, M.B., O'Boyle, G., et al. (2017). αEβ7 integrin identifies subsets of pro-inflammatory colonic CD4+ T lymphocytes in ulcerative colitis. J. Crohn's Colitis *11*, 610–620.

49. Kurmaeva, E., Lord, J.D., Zhang, S., Bao, J.R., Kevil, C.G., Grisham, M.B., and Ostanin, D.V. (2014). T cell-associated α4β7 but not α4β1 integrin is required for the induction and perpetuation of chronic colitis. Mucosal Immunol. *7*, 1354–1365.

50. Zhao, S., Fung-Leung, W.P., Bittner, A., Ngo, K., and Liu, X. (2014). Comparison of RNA-seq and microarray in transcriptome profiling of activated T cells. PLoS ONE *9*, e78644.

51. Lee, J.C., Lyons, P.A., McKinney, E.F., Sowerby, J.M., Carr, E.J., Bredin, F., Rickman, H.M., Ratlamwala, H., Hatton, A., Rayner, T.F., et al. (2011). Gene expression profiling of CD8+ T cells predicts prognosis in patients with Crohn disease and ulcerative colitis. J. Clin. Invest. *121*, 4170–4179.

52. Dalton, J.E., Cruickshank, S.M., Egan, C.E., Mears, R., Newton, D.J., Andrew, E.M., Lawrence, B., Howell, G., Else, K.J., Gubbels, M.J., et al. (2006). Intraepithelial γδ+ lymphocytes maintain the integrity of intestinal epithelial tight junctions in response to infection. Gastroenterology *131*, 818–829.

53. Lutter, L., Hoytema van Konijnenburg, D.P., Brand, E.C., Oldenburg, B., and van Wijk, F. (2018). The elusive case of human intraepithelial T cells in gut homeostasis and inflammation. Nat. Rev. Gastroenterol. Hepatol. *15*, 637–649.

54. Mayassi, T., and Jabri, B. (2018). Human intraepithelial lymphocytes. Mucosal Immunol. *11*, 1281–1289.

55. Bhat, P., Leggatt, G., Waterhouse, N., and Frazer, I.H. (2017). Interferon-γ derived from cytotoxic lymphocytes directly enhances their motility and cytotoxicity. Cell Death Dis. *8*, e2836.

56. Qiu, Y., and Yang, H. (2013). Effects of intraepithelial lymphocyte-derived cytokines on intestinal mucosal barrier function. J. Interferon Cytokine Res. *33*, 551–562.

57. Takashima, S., Martin, M.L., Jansen, S.A., Fu, Y., Bos, J., Chandra, D., O'Connor, M.H., Mertelsmann, A.M., Vinci, P., Kuttiyara, J., et al. (2019). T cell-derived interferon-γ programs stem cell death in immune-mediated intestinal damage. Sci. Immunol. *4*, eaay8556.

58. Youakim, A., and Ahdieh, M. (1999). Interferon-gamma decreases barrier function in T84 cells by reducing ZO-1 levels and disrupting apical actin. Am. J. Physiol. *276*, G1279–G1288.

59. Tang, F., Sally, B., Lesko, K., Discepolo, V., Abadie, V., Ciszewski, C., Semrad, C., Guandalini, S., Kupfer, S.S., and Jabri, B. (2015). Cysteinyl leukotrienes mediate lymphokine killer activity induced by NKG2D and IL-15 in cytotoxic T cells during celiac disease. J. Exp. Med. *212*, 1487–1495.

60. Boland, B.S., He, Z., Tsai, M.S., Olvera, J.G., Omilusik, K.D., Duong, H.G., Kim, E.S., Limary, A.E., Jin, W., Milner, J.J., et al. (2020). Heterogeneity and clonal relationships of adaptive immune cells in ulcerative colitis revealed by single-cell analyses. Sci. Immunol. *5*, eabb4432.

61. Corridoni, D., Antanaviciute, A., Gupta, T., Fawkner-Corbett, D., Aulicino, A., Jagielowicz, M., Parikh, K., Repapi, E., Taylor, S., Ishikawa, D., et al. (2020). Single-cell atlas of colonic CD8+ T cells in ulcerative colitis. Nat. Med. *26*, 1480–1490.

62. Cheroutre, H., Lambolez, F., and Mucida, D. (2011). The light and dark sides of intestinal intraepithelial lymphocytes. Nat. Rev. Immunol. *11*, 445–456.

63. Mayassi, T., Ladell, K., Gudjonson, H., McLaren, J.E., Shaw, D.G., Tran, M.T., Rokicka, J.J., Lawrence, I., Grenier, J.C., van Unen, V., et al. (2019). Chronic inflammation permanently reshapes tissue-resident immunity in celiac disease. Cell *176*, 967–981.e19.

64. Danese, S., Colombel, J.-F., Lukas, M., Gisbert, J.P., D'Haens, G., Hayee, B., Pannacione, R., Kim, H.-S., Reinisch, W., Tyrrell, H., et al. (2020). Etrolizumab versus infliximab for treating patients 35RASpberry809 with moderately to severely active ulcerative colitis: results from the phase III GARDENIA study. United European Gastroenterol. J. *8*, 1258–1275.

65. Wu, T.D., and Nacu, S. (2010). Fast and SNP-tolerant detection of complex variants and splicing in short reads. Bioinformatics *26*, 873–881.

66. Phipson, B., Lee, S., Majewski, I.J., Alexander, W.S., and Smyth, G.K. (2016). Robust hyperparameter estimation protects against hypervariable genes and improves power to detect differential expression. Ann. Appl. Stat. *10*, 946–963.

67. Stuart, T., Butler, A., Hoffman, P., Hafemeister, C., Papalexi, E., et al. (2019). Comprehensive Integration of Single-Cell Data. Cell *177*, 1888–1902.

68. Bueno, R., Stawiski, E.W., Goldstein, L.D., Durinck, S., De Rienzo, A., Modrusan, Z., Gnad, F., Nguyen, T.T., Jaiswal, B.S., Chirieac, L.R., et al. (2016). Comprehensive genomic analysis of malignant pleural mesothelioma identifies recurrent mutations, gene fusions and splicing alterations. Nat. Genet. *48*, 407–416.

69. Orozco, L.D., Chen, H.H., Cox, C., Katschke, K.J., Jr., Arceo, R., Espiritu, C., Caplazi, P., Nghiem, S.S., Chen, Y.J., Modrusan, Z., et al. (2020). Integration of eQTL and a single-cell atlas in the human eye identifies causal genes for age-related macular degeneration. Cell Rep. *30*, 1246–1259.e6.

70. Kumar, M.S., Slud, E.V., Okrah, K., Hicks, S.C., Hannenhalli, S., and Corrada Bravo, H. (2018). Analysis and correction of compositional bias in sparse sequencing count data. BMC Genomics *19*, 799.

71. Law, C.W., Chen, Y., Shi, W., and Smyth, G.K. (2014). voom: Precision weights unlock linear model analysis tools for RNA-seq read counts. Genome Biol. *15*, R29.

72. Benjamini, Y., Drai, D., Elmer, G., Kafkafi, N., and Golani, I. (2001). Controlling the false discovery rate in behavior genetics research. Behav. Brain Res. *125*, 279–284.

STAR★METHODS

KEY RESOURCES TABLE

REAGENT or RESOURCE	SOURCE	IDENTIFIER
**Antibodies**		
APC anti-mouse CD45 (clone 30-F11)	BioLegend	Catalog #: 103112
PE/Cy7 anti-mouse TCRβ (clone H57-597)	BioLegend	Catalog #: 109222
APC/Cy7 anti-mouse TCRvα2 (clone B20.1)	BioLegend	Catalog #: 127818
PerCP/Cy5.5 anti-mouse CD8 (clone 53-6.7)	BioLegend	Catalog #: 100734
APC/Cy7 anti-mouse CD4 (clone GK1.5)	BioLegend	Catalog #: 100414
BV510 anti-mouse CD62L (clone MEL14)	BioLegend	Catalog #: 104441
BUV395 anti-mouse CD44 (clone 1M7)	BD Bioscience	Catalog #: 740215
APC/Cy7 anti-mouse MHCII (clone M5/114.15.2)	BioLegend	Catalog #: 107628
PE/Cy7 anti-mouse CD11c (clone N418)	BioLegend	Catalog #: 117318
FITC anti-mouse/human CD11b (clone M1/70)	BioLegend	Catalog #: 101206
PE anti-mouse FOXP3 (clone MF14)	BioLegend	Catalog #: 126404
FITC anti-mouse IFN-γ (clone XMG1.2)	BioLegend	Catalog #: 505806
PE anti-mouse IL-17A (clone TC11-18H10.1)	BioLegend	Catalog #: 506904
APC anti-mouse TNF-α (clone TN3-19.12)	BioLegend	Catalog #: 506108
PerCP/Cy5.5 anti-mouse CD103 (clone M290)	BD Bioscience	Catalog #: 563637
APC anti-mouse CD103 (clone 2E7)	BioLegend	Catalog #: 121414
Anti-Laminin antibody produced in rabbit	Sigma-Aldrich	SKU no: L9393
Rat anti-mouse E-cadherin (clone ECCD2)	Thermo fisher	Catalog #: 13-1900: RRID: AB_2533005
Rabbit polyclonal anti-EpCAM	Abcam	ab71916
Goat anti-mouse LYVE-1 (R&D systems)	R&D systems	Catalog #: AF2125
TruStain FcX (anti-mouse CD16/32) Antibody (clone 93)	BioLegend	Catalog #: 101320
BV786 anti-human CD45 (clone HI30)	BioLegend	Catalog #: 304048
Alexa Fluor 700 anti-human CD8 (clone HIT8a)	BioLegend	Catalog #: 300920
PE/Cy7 anti-human CD4 (clone RPA-T4)	BioLegend	Catalog #: 300512
PerCP/Cy5.5 anti-human CD103 (clone Ber-ACT8)	BioLegend	Catalog #: 350226
PE anti-human/mouse β7 (clone FIB504)	BioLegend	Catalog #: 321204
BV605 anti-human TCRαβ (clone IP26)	BD Bioscience	Catalog #: 745088
BV711 anti-human CD25 (clone M-A251)	BioLegend	Catalog #: 356138
Pacific Blue anti-human FOXP3 (clone 259D)	BioLegend	Catalog #: 320216
APC anti-mouse/human Helios (clone 22F6)	BioLegend	Catalog #: 137222
FITC anti-human IFN-γ (clone B27)	BioLegend	Catalog #: 506504
PE anti-human IL-17A (clone N49-4653)	BD Bioscience	Catalog #: 560486
APC anti-human TNF-α (clone MAb11)	BioLegend	Catalog #: 502912
PE/Cy7 anti-human CD49a (clone TS2/7)	BioLegend	Catalog #: 328312
PE anti-human IL-15Rα (clone JM7A4)	BioLegend	Catalog #: 330208
BV605 anti-human NKG2D (clone 1D11)	BioLegend	Catalog #: 320832
BV650 anti-human IL-7Rα (clone A019D5)	BioLegend	Catalog #: 351326
Pacific Blue anti-human KLRB1 (clone HP-3G10)	BioLegend	Catalog #: 339914
Alexa Fluor 647 anti-human/mouse Granzyme B (clone GB11)	BioLegend	Catalog #: 515406
FITC anti-human Granzyme A (clone CB9)	BioLegend	Catalog #: 507204
PE anti-human Perforin (clone B-D48)	BioLegend	Catalog #: 353304
*InVivo*mAb anti-mouse IL-10R (clone 1B1.3A)	Bio X Cell	Catalog #: BE0050 RRID: AB_1107611
*InVivo*mAb anti-mouse CD103 (Clone M290)	Bio X Cell	Catalog #: BE0026 RRID: AB_1107570

(*Continued on next page*)

CellPress
OPEN ACCESS

Continued

REAGENT or RESOURCE	SOURCE	IDENTIFIER
anti-E-cadherin antibody (Clone ECCD-2)	Invitrogen	Catalog # 13-1900
anti-β7 (clone FIB504)	This paper	RRID:AB_2892125
anti-α4β7 (clone DATK32)	This paper	RRID: AB_2892121
**Bacterial and virus strains**		
Helicobacter hepaticus Fox et al.	ATCC	Strain no: 51448
**Biological samples**		
Diverticulitis or ulcerative colitis tissue samples (surgical resections)	Mayo Clinic	Mayo Clinic Institutional Review Board (IRB 10-006628)
Healthy or IBD patient intestinal tissue biopsies	Stanford	Stanford Institutional Review Board (IRB protocol 28427)
Healthy intestinal tissue biopsies	UCSF	UCSF Institutional Review Board (IRB 10-00263 and 10-01218)
**Chemicals, peptides, and recombinant proteins**		
Ovalbumin (257-264) chicken	Sigma-Aldrich	SKU no: S7951
Cholera toxin from *Vibrio cholerae*	Sigma-Aldrich	SKU no: C8052
FTY720	Sigma-Aldrich	SKU no: SML0700
**Critical commercial assays**		
Foxp3 / Transcription Factor Staining Buffer Set	eBioscience	Catalog number: 00-5523-00
BD Cytofix/Cytoperm Fixation/Permeabilization kit	BD Bioscience	Catalog number: 554714
Cell Activation Cocktail (with Brefeldin A)	BioLegend	Catalog number: 423304
CD8α^{+} T Cell Isolation Kit, mouse	Miltenyi Biotec	Order no: 130-104-075
Lamina Propria Dissociation Kit, mouse	Miltenyi Biotec	Order no: 130-097-410
Zombie NIR^{TM} Fixable Viability Kit	BioLegend	Catalog number: 423106
Chromium Single Cell 3′ GEM, Library & Gel Bead Kit v3	10x Genomics	PN-1000075
Chromium Single Cell B Chip Kit	10x Genomics	PN-1000153
Chromium i7 Multiplex Kit	10x Genomics	PN-120262
Bioanalyzer High Sensitivity DNA kit	Agilent Technologies	Part number: 5067-4626 & 5067-4627
Kapa Library Quantification Kit	Roche Diagnostics	Catalog number: 07960409001
**Deposited data**		
Bulk RNA-sequencing of Crohn's disease clinical trial samples	This paper	GEO: GSE152316
Bulk RNA-sequencing of sorted colonic CD103+ and CD103- T cells	This paper	GEO: GSE152320
Single cell RNA-sequencing of healthy colonic immune cells	This paper	GEO: GSE152290
RNA-sequencing of individual sorted colonic T cells	This paper	GEO: GSE152306
RNA-sequencing of colonic biopsies from vedolizumab-treated patients	Arijs et al.[39]	GEO: GSE73661
Human reference genome NCBI build 38, GRCh38	Genome Reference Consortium	https://www.ncbi.nlm.nih.gov/projects/genome/assembly/grc/human/
GENCODE (v27)	GENCODE Project	https://www.gencodegenes.org/human/release_27.html
**Experimental models: Organisms/strains**		
Mouse: C57BL/6	Charles River	Strain code: 027
C57BL/6NTac	Taconic Biosciences	Model #: B6-F
Tg(Tg(CAG-KikGR)33Hadj/J-KikGR)33Hadj/J	The Jackson Laboratory	Stock No: 013753
C57BL/6-Tg(TcraTcrb)1100Mjb/J	The Jackson Laboratory	Stock No: 003831
B6.Cg-*Gt(ROSA)26Sor*$^{tm14(CAG-tdTomato)Hze}$/J	The Jackson Laboratory	Stock No: 007914
B6.129P2(Cg)-*Cdh1*^{tm1Cle}/J	The Jackson Laboratory	Stock No: 016933

(*Continued on next page*)

Continued		
REAGENT or RESOURCE	SOURCE	IDENTIFIER
B6.Cg-Tg(CD4-cre)1Cwi N9	Taconic Biosciences	Model #: 4196
KikGR-OT-1	This paper	
TdTomato-OT-1	This paper	
Software and algorithms		
R (v 3.5.1)	The R Project	http://www.r-project.org
GSNAP (v 2013-11-01)	Wu and Nacu[65]	http://research-pub.gene.com/gmap/
HTSeqGenie R package (v 4.12.0)	Pau and Reeder	https://doi.org/doi:10.18129/B9.bioc.HTSeqGenie
Limma R package (v 3.38.3)	Phipson et al.[66]	https://doi.org/doi:10.18129/B9.bioc.limma
Seurat (v3.0)	Stuart et al.[67]	https://cran.r-project.org/web/packages/Seurat/index.html
GSDecon R package (v 2.4.39)	Bueno et al.[68]	https://github.com/JasonHackney/GSDecon
Imaris 9.2.1	Bitplane, an Oxford Instruments company	https://imaris.oxinst.com/versions/9-2
GraphPad Prism v.7 and v.8	GraphPad Software	https://www.graphpad.com/scientific-software/prism/
FlowJo v.9 and v.10	FlowJo	https://www.flowjo.com/
ImageJ v1.52	NIH	https://imagej.net/downloads

RESOURCE AVAILABILITY

Lead contact

Further information and requests for resources and reagents should be directed to and will be fulfilled by the lead contact, Tangsheng Yi (yi.tangsheng@gene.com).

Materials availability

All unique/stable reagents generated in this study are available from the lead contact, Tangsheng Yi, with a completed Materials Transfer Agreement.

Data and code availability

All RNA-sequencing data generated during this study are available at the Gene Expression Omnibus (GEO) under accession number GSE152321. Vedolizumab treatment data were downloaded from GEO using accession number (GEO: GSE73661).

EXPERIMENTAL MODEL AND SUBJECT DETAILS

Mice

All of the mice used in this study were bred and maintained at Genentech under specific pathogen-free conditions, and were cared for in accordance with institutional guidelines with review and approval by the Genentech Institutional Animal Care and Use Committee. Female C57BL/6 wild-type mice were from Charles River Laboratories or Taconic Biosciences, and were used at age 6-8 weeks for studies. KikGR transgenic mice[27] (Tg(CAG-KikGR)33Hadj/J, 013753), and E-cadherin-mCFP mice (B6.129P2(Cg)-*Cdh1*^{tm1Cle}/J, 016933) were obtained from the Jackson Laboratories and bred at Genentech. OT1-TCR transgenic mice (Tg(TcraTcrb)1100Mjb/J, 003831), and LSL.tdTomato mice (B6.Cg-Gt(ROSA)26Sor$^{tm14(CAG\text{-}tdTomato)Hze/J}$, 007914) were obtained from the Jackson Laboratories and were used for breeding. CD4.Cre (B6.Cg-Tg(CD4-cre)1CwiN9, 4196) mice were obtained from Taconic and were used for breeding. KikGR-OT-1 and TdTomato-OT-1 transgenic mice were generated at Genentech by crossing OT-1-TCR transgenic mice with KikGR transgenic mice or LSL.tdTomato mice, respectively. Female KikGR, E-cadherin-mCFP, KikGR-OT-1 and TdTomato-OT-1 transgenic mice were used at age 6-8 weeks for studies.

Patient samples

Analysis of patient samples included three distinct cohorts independent of the etrolizumab clinical study NCT02394028. Biopsies from healthy subjects undergoing routine flexible sigmoidoscopy or endoscopy as part of their clinical care at UCSF (IRB 10-00263 and 10-01218; UCSF Institutional Review Board) were processed for single cell analysis or flow cytometry as described below. IBD patients undergoing routine endoscopy as part of their clinical care at Stanford were enrolled in a prospective, observational registry study (IRB 28247; Stanford Institutional Review Board) to donate biopsies for flow cytometry. For single cell

analysis of sorted αE^{+} and αE^{-} T cells, tissue samples from patients undergoing intestinal resections as part of their clinical care at the Mayo Clinic. into an observational study (IRB 10-006628; Mayo Clinic Institutional Review Board). All patients provided consent in accordance with Institutional Review Board guidance for each institution as noted above. Patient characteristics are shown in Table S1.

Etrolizumab clinical study in CD

Moderately to severely active CD patients with a CDAI or 220-480 and a centrally read SES-CD score of ≥ 7 (≥4 for isolated ileitis) who had previously been treated with corticosteroids, immunosuppressants or anti-TNF were enrolled in an exploratory cohort of 350 patients within the BERGAMOT etrolizumab study (ClinicalTrials.gov: NCT02394028). Briefly, patients underwent endoscopy as part of their screening visit and baseline biopsies were taken in the ileum or the colon for analysis prior to randomization. Patients were then randomized into placebo or etrolizumab cohorts at a 1:4 ratio with subcutaneous administration at 0, 2, 4, 8 and 12 weeks. Following the induction period of 14 weeks, patients underwent a second ileocolonoscopy and follow-up biopsies were obtained from the ileum or colon and placed directly into RNAlater (QIAGEN). Biopsy samples were scored as being from active or inactive segments using the cut-off of colonic SES-CD ≥ 7 for colonic biopsies and ileal SES-CD ≥ 4 for ileal biopsies. Patient characteristics are shown in Table S2.

METHOD DETAILS

Blocking antibodies for murine studies

Anti-β7 (FIB504) and anti-α4β7 (DATK32) antibodies were ordered from ATCC and their sequences were individually cloned from hybridomas. Antigen-binding domain sequences from the parent hybridomas were fused to mouse IgG1 Fc in a pRK expression vector and transfected into HEK293 cells. Transfected supernatants were purified on a HiTrap column (GE healthcare) with Mabselect Sure resin (GE healthcare) with a phosphate-buffered saline (PBS) loading buffer. Antibodies were eluted with 0.1 M citrate (pH 3.0) and neutralized with 3 M Tris, pH 8.0, to a final pH of ~7.0 prior to dialysis against PBS, pH 7.2. Each antibody was run on a Superdex S200 10/300 GL size exclusion column (SEC) (GE Healthcare) using PBS, pH 7.2, load buffer at a flow rate of 1 mL/min (30 cm/h) to remove any aggregates. Pooled fractions were filtered using a 0.2 mm filter and the final antibody preparation was assessed by analytical SEC carried out with a TSK-GEL, Super SW3000, 4.6 mm × 30 cm, 4 mm (Tosoh Bioscience) column using a Dionex Ultimate 3000 system (Thermo Fisher Scientific) to confirm > 95% homogeneity of monomeric antibody. Anti-αE blocking antibody (clone number M290) was from BioXcell and anti-E-cadherin antibody (clone number ECCD-2) was from Invitrogen.

Flow cytometry antibodies and staining

Cell suspension was washed twice in staining buffer (PBS with 2% of fetal bovine serum), blocked with TruStain FcX (anti-mouse CD16/32, Biolegend) for 5mins at room temperature, and stained with indicated fluorescence conjugated antibodies below for 30mins. For intranuclear staining, cells were fixed, permeabilized and stained using the Foxp3/Transcription Factor Staining Kit according to the manufacturer's instructions (eBioscience). For intracellular cytokine staining, cells were stimulated with the Cell Activation Cocktail with Brefeldin A (BioLegend) for 5 hours, followed by fixation, permeabilization and staining using the Cytofix/Cytoperm kit (BD Bioscience). Fluorochrome-conjugated anti-mouse antibodies used are as follows: CD45 (30-F11), TCRβ (H57-597), CD8 (53-6.7), CD4 (GK1.5), CD62L (MEL14), CD44 (1M7), MHCII (M5/114.15.2), CD11c (N418), CD11b (M1/70) IFN-γ (XMG1.2), IL-17A (TC11-18H10.1), TNF-α (TN3-19.12), CD103 (M290, 2E7), Zombie NIR (live/dead, Biolegend). Fluorochrome-conjugated anti-human antibodies used are as follows: CD45 (HI30), CD8 (HIT8a), CD4 (RPA-T4), CD103 (Ber-ACT8), β7 (FIB504), TCRαβ (IP26), CD25 (M-A251), Foxp3 (259D), Helios (22F6), IFN-γ (B27), IL-17A (N49-4653), TNF-α (MAb11), CD49a (TS2/7), IL-15Rα (JM7A4), NKG2D (1D11), IL-7Rα (A019D5), KLRB1 (HP-3G10), Granzyme B (GB11), Granzyme A (CB9), Perforin (B-D48).

Murine surgical photoconversion models of T cell trafficking

Photoconversion of mLN or specified intestinal segment was performed during survival surgeries carried out in a sterile environment under proper anesthesia. Following shaving of the surgical site, a small abdominal incision of ~1 cm allowed the cecum to be gently extruded to localize mLN, colon, and/or small intestine. A piece of sterile foil with a 5-mm hole was used to surround the tissue of interest, e.g., mLN, and limit exposure of surrounding tissue to the photoconverting light. For ileum photoconversion, regions with Peyer's patches (PPs) was avoided. A silver LED (Prizmatix) with an aperture polymer optical fiber (1.5 mm core diameter) and a lens focuser was used as the violet light source (415 nm) to treat the target tissue as indicated. Each exposed area was treated with violet light for a total period of 5 minutes then extruded organs were returned to the abdominal cavity and the incision was sutured closed. Anti-integrin antibodies (250 μg/mouse) were administered intraperitoneally (IP) 3 hours prior to photoconversion and analysis was performed 16 hours post photoconversion.

T cell adoptive transfer and oral antigenic challenge

KikGR^{+}OT-1^{+} green fluorescent CD8^{+} T cells were enriched from splenocytes using the MACS CD8^{+} T cells isolation kit (Miltenyi Biotech) according to the manufacturer's instructions. Each C57BL/6 wild-type recipient mouse received 5 × 10^{6} KikGR^{+}OT-1^{+} T cells intravenously (IV) and were then orally challenged with 150 μg Cholera toxin:OVA (Sigma) at a 1:25 molar ratio the following

day. Three days post-challenge, 250 μg of anti-integrin antibodies were administered IP, the mice were rested overnight (16 hours) then sacrificed for analysis. Alternatively, anti-integrin antibodies (250 μg/mouse) were administered IP 3 hours prior to photoconversion of small intestine and analysis was performed 16 hours post photoconversion.

Isolation and quantification of murine IEL and LP lymphocytes

Intestinal IEL and LP lymphocytes were prepared using the MACS lamina propria dissociation kit (Miltenyi Biotech). Briefly, the colon or small intestine was cut into 1-cm pieces following removal of PPs, then incubated in Hank's Balanced Salt Solution (HBSS) (Ca^{2+} and Mg^{2+} free) containing EDTA and DTT to isolate IELs. Following removal of IELs, the remaining tissue was transferred to HBSS (with Ca^{2+} and Mg^{2+}) supplemented with collagenase to release LP lymphocytes. Isolated IEL and LP lymphocytes were washed in PBS and counted prior to staining for flow cytometry. For photoconversion studies, the percentage of $KikR^+$ cells within the live $TCR\beta^+CD8^+$ cell gate was normalized to the percent of $KikR^+$ non-migratory DCs in the mLN to account for differences in photoconversion efficiency between experiments.

Intra-vital two-photon microscopy live imaging

T cell adoptive transfer and oral antigenic challenge was performed as described above but using $tdTomato^+OT\text{-}1^+$ $CD8^+$ T cells adoptively transferred into E-cadherin-mCFP recipient mice to enable intravital imaging. Intravital microscopy was performed on anesthetized mice with the ileum exposed and opened 1 cm longitudinally along the anti-mesenteric border. The incision site was placed far away from the vasculature to ensure continuous blood supply. The mucosal surface was placed against a customized stage with a moistened PBS pad to prevent dehydration. The tissue was immobilized on a stage using a plastic ring with pinholes and a coverslip was placed on top of the mucosal surface. The luminal side of the ileum was imaged using a two-photon laser-scanning microscope (Ultima *In Vivo* Multiphoton Microscopy System; Bruker Technologies) using a 16X objective (Olympus) and dual Ti:sapphire lasers (MaiTai and Insight lasers, Spectra-Physics;) tuned to 890 nm and 1020 nm. Analysis was performed using Imaris 9.2.1 (Bitplane; an Oxford Instruments company).

Immunofluorescence and confocal microscopy

For confocal microscopy, T cell adoptive transfer and oral antigenic challenge was performed as described above. Upon sacrifice of recipient mice, the ileal portion of the small intestine was excised and prepared as a "swiss roll." Tissues were fixed in 4% paraformaldehyde for 6-8 hours or in Cytofix/Cytoperm Buffer (BD Bioscience) for 2-4 hours for lymphatics staining, and then dehydrated in 30% sucrose overnight prior to embedding in optimal cutting temperature (OCT) freezing media. Cryosections (8-10 μm) were stained with the following primary antibodies: Laminin (Sigma-Aldrich, L9393), E-cadherin (Thermo fisher, ECCD2), EpCAM (Abcam) and LYVE-1 (R&D systems). All images were acquired using a Leica TCS SPE upright confocal microscope. Frequency of $tdTomato^+$ cells in $LYVE1^+$ lymphatics was blindly quantified from 100 villi across tissue sections and a mean percentage of 100 villi was used for each individual mouse.

Helicobacter hepaticus anti-IL10R induced colitis model

Female C57BL/6 mice (Taconic) at 5-6 weeks old were treated weekly with anti-IL10R antibody (1 mg/ mouse administered IP) (BioXcell). Oral administration of $\sim 1.2 \times 10^9$ colony-forming units (CFU) *Helicobacter hepaticus* (ATCC 51448) was performed on two consecutive days as previously described,[35] and mice were monitored three times per week for signs of colitis, such as watery stool. Following onset of disease symptoms, colitic mice were sacrificed and cells were isolated from the intestine as described above.

Preparation of cells from patient tissue samples for flow cytometry

Patient resection samples from the Mayo clinic and patient colonic biopsies from UCSF were shipped on ice in RPMI 1640 containing 10% FBS and antibiotics. Frozen biopsies from Stanford were thawed in a 37°C water bath immediately prior to processing. For resection tissue, the mucosa layer was separated from the serosal layer and cut into small pieces. Tissue samples were washed in HBSS (w/o Mg^{2+}) and then transferred to 37°C prewarmed digestion buffer (DMEM/F12 50:50, 10% FBS, 15mM HEPES, PenStrep, 0.35mg/mL Collagenase D, 0.5mM DTT) and incubated in a 37°C water bath for 10 minutes with agitation by shaking every 5 minutes. Cells released from the tissue into the supernatant were removed and transferred to the wash buffer (DMEM/F12 50:50, 10% FBS, 15mM HEPES, PenStrep). Following three rounds of digestion, pooled cells were spun down, counted and stained as described above for flow cytometry analysis or sorting.

Preparation of tissue samples for RNA-seq

$CD45^+TCR\alpha\beta^+$ $TCR\gamma^-$ αE^+ and αE^- single cells were sorted directly into RLT lysis buffer (QIAGEN) with β-mercaptoethanol. RNA was isolated from sorted cells using the QIAGEN RNeasy Mini Kit (QIAGEN) according to the manufacturer's instructions. Tissues were digested into single cell suspension and sorted by FACS into 3 populations: $CD45^+Epcam^-$, $CD45^-Epcam^+$, and $CD45^-Epcam^-$. Sort time was capped at 2 hours. Sample processing for single-cell RNA-seq was done using Chromium Single Cell 3′ Library and Gel bead kit v3 following manufacturer's guide (10x Genomics). The cell density and viability of single-cell suspension were determined by Vi-CELL XR cell counter (Beckman Coulter). All of the processed samples had a very high percentage of viable cells. The cell

density was used to infer the volume of single cell suspension needed in the reverse transcription (RT) master mix, aiming to achieve ~6,000 cells per sample. cDNAs and libraries were prepared following the manufacturer's user guide (10x Genomics). Libraries were profiled by Bioanalyzer High Sensitivity DNA kit (Agilent Technologies) and quantified using Kapa Library Quantification Kit (Kapa Biosystems). Each library was sequenced in one lane of HiSeq4000 (Illumina) following the manufacturer's sequencing specification (10x Genomics).

Biopsies from the etrolizumab clinical study were thawed, removed from RNAlater, homogenized with 0.1 mm glass beads and RNA was purified using QIAGEN All-prep 96 kits. Quantity and quality of the RNA was assessed with a NanoDrop 8000 (Thermo Scientific) and Bioanalyzer, respectively. Sequence libraries were prepared from 0.1 μg of RNA using TruSeq Stranded Total RNA Library Prep kit (Illumina) according to the manufacturer's instructions. Size of the libraries was confirmed using 4200 TapeStation and High Sensitivity D1K screen tape (Agilent Technologies) and their concentration was determined by a qPCR-based method using a Library Quantification Kit (KAPA). Initially, 387 libraries were multiplexed and sequenced on Illumina HiSeq4000 to generate 30M single end 50 base pair reads. Samples (n = 255) with a minimum of 20 million high quality reads were determined to be acceptable for further analysis, while samples below that threshold (n = 132) underwent additional library preparation and sequencing using a NovaSeq 6000 S2 flow cell (100 cycles), with a total read length of 1x50bp. Samples with little or poor quality RNA (n = 29) were excluded from further sequencing and analysis. Analysis of the study combined results from both sequencing runs.

Single cell RNA-seq analysis

Single cell RNA-seq data collected using 10x Genomics were processed as previously described,[69] with the following modifications: only exonic counts were used for determining gene expression counts, and barcodes with $\geq$ 2000 exonic UMIs with < 8% of UMIs coming from mitochondrial genes were considered to contain viable cells. For downstream analyses, the Seurat package (v 3.0) was used with default values to normalize transcript counts, and CCA was used to align expression data across individual donors. Graph-based clustering identified groups of cells at relatively low resolution corresponding to broad cell types in the $CD45^+$ sorted population. We repeated the procedure within the T cell population to identify subtypes of T cells. A Wilcoxon test was used to identify gene expression markers for each cell type. If < 30 genes with $\geq$ 1.5-fold higher expression were found in a cluster, it was merged with the most similar cluster of cells. In this way, we defined broad subsets of T cells that were manually annotated using marker gene lists specific to each cell cluster. The most differential genes for each cluster, including genes with an FDR < 0.01, were used to define signatures of at most 50 genes for each T cell subtype. For full length RNA-seq of individual sorted αE^+ and αE^- T cells, wrench was used to normalize the raw counts,[70], which were then transformed using voom and differential expression was determined using the limma package.[71,66]

Bulk RNA-seq analyses

For bulk RNA-seq analysis, we used custom scripts written in the R programming language and packages from the Bioconductor project. Raw RNA-seq reads were processed using the HTSeqGenie R package, as previously described.[69] Briefly, RNA-seq data was aligned to the reference human genome (GRCh38) and reads that uniquely matched to exons of gene models present in the GENCODE basic annotation set (v. 27) were counted. Protein coding genes and immune (immunoglobulin, TCR) constant regions were included in the analysis. Genes were included in the analysis if they had > 10 reads in > 31 samples. For all differential expression analyses, we again used the voom transformation method coupled with limma-based linear modeling[71,66] and adjusted p values for multiple testing using the Benjamini-Hochberg procedure[72], unless otherwise stated. Signature scores were calculated as detailed in[68] using the GSDecon package (https://github.com/JasonHackney/GSDecon).

Etrolizumab-specific changes were calculated as a log2-fold change in gene expression (count per million, CPM) values between week 14 and screening biopsy samples from the same bowel segment. A t test was used to determine the significance of the log2-fold between etrolizumab-treated samples and placebo-treated samples. Only genes that were significant in the transcriptome-wide analysis were considered in this analysis. The same method was used to assess changes in T cell signature scores.

Patient metadata was included as co-variates in the analysis of biopsies from patients enrolled in the clinical study of etrolizumab. To identify gene expression modulated by etrolizumab treatment, we used a linear model that included treatment arm (etrolizumab v. placebo), prior anti-TNF incomplete response, biopsy location (colon v. ileum), time of biopsy (screen v. post-induction) and the interaction between treatment arm and visit. Genes were considered significantly modulated by etrolizumab if they had an absolute fold change $\geq$ 1.5, at an FDR of < 0.05 in the combined colonic and ileal analysis, and were nominally significant (unadjusted p value < 0.05) in both the colonic- and ileal-only analyses. Samples were included in the analysis of etrolizumab treatment effects if RNA-seq data was available for both time points and if the biopsy was taken from an endoscopically active region at the screening visit. To determine differential gene expression in active v. inactive bowel segments, we used a linear model that included covariates for prior anti-TNF incomplete response and anatomic location of the biopsy. Only screening visit biopsies were included in this analysis.

QUANTIFICATION AND STATISTICAL ANALYSIS

Statistical methods and software packages used are described in detail in the Method details. Statistical analyses for both bulk and single cell RNA-sequencing were performed using R software (version 3.5.1) and R packages as described in the Methods details and legends for Figures 5 and 6. Exclusion criteria for analyses are described in the Method details.

GraphPad Prism was utilized for statistical analysis on Figures 1, 2, 3, 4, 5, and 6. Statistical details of experiments can be found in figure legends, including the statistical tests used and value and definition of n.

Differences were considered to be statistically significant when $p < 0.05$. For biological experiments, sample sizes were determined based on previous experience with similar experiments.

ADDITIONAL RESROUCES

Further information about sample preparation, data collection, or data processing is described in the Method details and can also be directed to the lead contact. Web resources containing the clinical trial design and enrollment criteria can be found in http://ClinicalTrials.gov. Registry number for BERGAMOT study is NCT02394028.

Cell Reports Medicine, Volume *2*

Supplemental information

Dual targeting of lymphocyte homing and retention through α4β7 and αEβ7 inhibition in inflammatory bowel disease

Bingbing Dai, Jason A. Hackney, Ryan Ichikawa, Allen Nguyen, Justin Elstrott, Luz D. Orozco, Kai-Hui Sun, Zora Modrusan, Alvin Gogineni, Alexis Scherl, John Gubatan, Aida Habtezion, Monika Deswal, Ma Somsouk, William A. Faubion, Akiko Chai, Zaineb Sharafali, Azra Hassanali, Young S. Oh, Swati Tole, Jacqueline McBride, Mary E. Keir, and Tangsheng Yi

Supplemental Figure and Figure legend

Figure S1

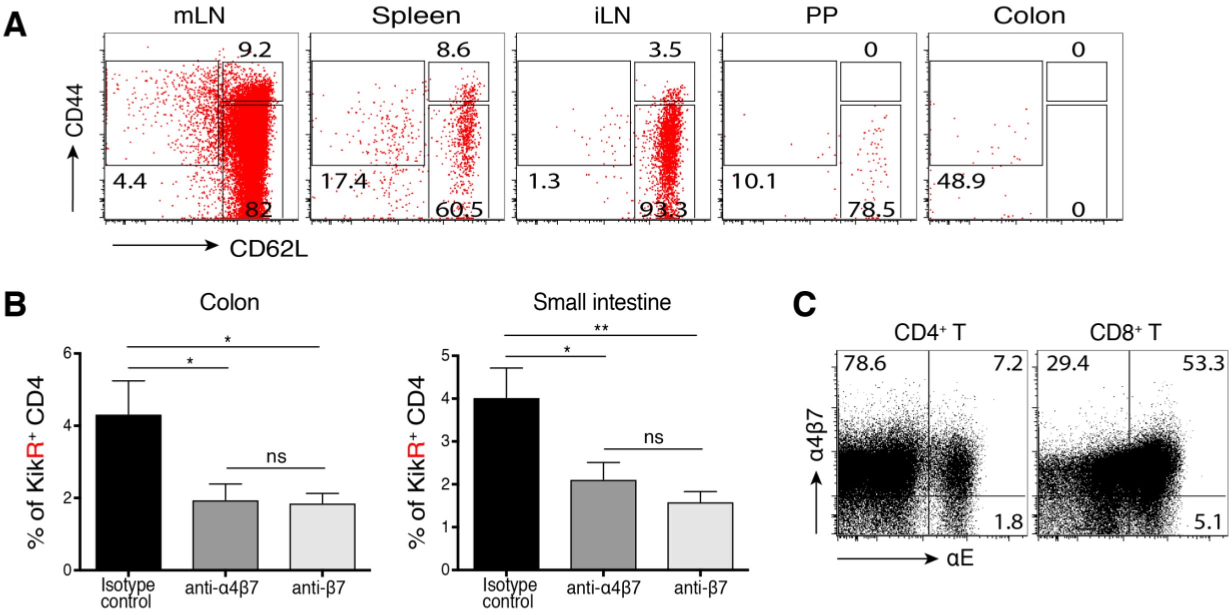

Figure S1. Tissue distribution and surface integrin expression of T cells following surgical photoconversion. Related to Figure 1. (**A**) Expression of CD44 and CD62L on KikR$^+$ CD8$^+$ T cells from lymphoid and gut tissues 16 hours after mLN photoconversion. (**B**) Frequency of photo-converted KikR$^+$ cells among CD45$^+$TCRβ$^+$CD4$^+$ T cells in colon (**left**) and small intestine (**right**). KikGR transgenic mice were treated with isotype or anti-integrin antibodies followed by surgical photoconversion of mLN. Small intestine and colon tissue were taken 16 hours post-photoconversion for flow cytometry analysis. Bar graph shows mean ± standard error (SEM). n=6-8 mice combined from 2 independent experiments where *=p<0.05, **=p<0.01, ***=p<0.001 by one-way ANOVA with Tukey's multiple comparison post-test. (**C**) Flow cytometry analysis of α4β7 and αE expression on gated CD4$^+$ or CD8$^+$ T cells from mLN. One representative plot is shown of three mice analyzed.

Figure S2

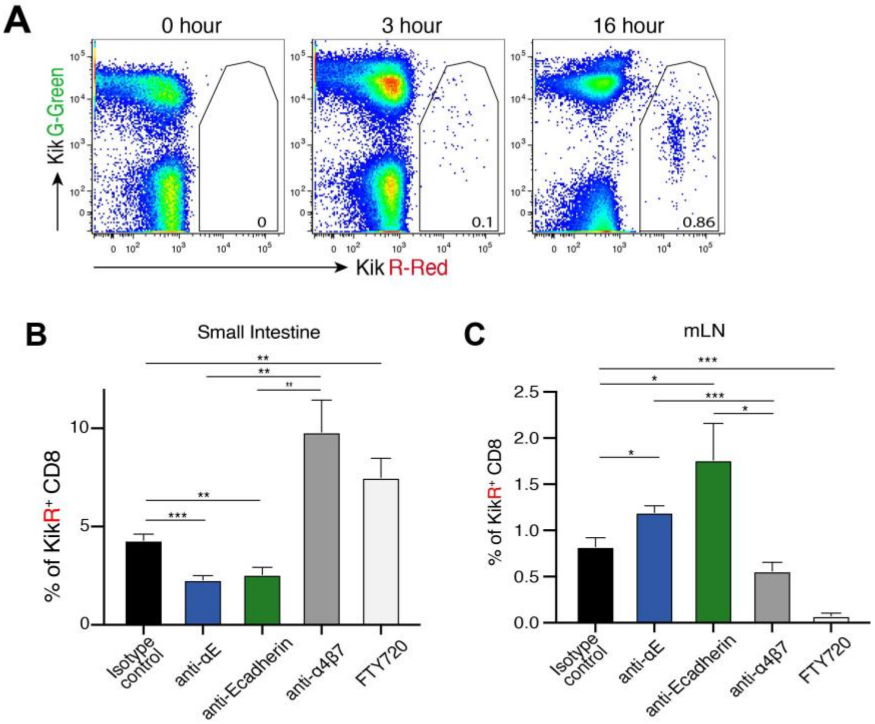

Figure S2. T cells photoconverted in the small intestine emigrate to the draining lymph node. Related to Figure 4. KikGR^{+} OT-1 cells were adoptively transferred into WT mice, then recipient mice were orally gavaged with cholera toxin:OVA. Three days post immunization, isotype or anti-integrin antibodies were administered followed by surgical photoconversion of a segment of the small intestine. After photo-conversion, small intestine and mLN were collected and cells were isolated and stained for flow cytometry analysis. (**A**) Representative FACS plot of mLN at 0, 3 and 16 hours following small intestine photostamping. (**B**) Percentage of KikR^{+} OT-1 cells in the small intestine (**B**) and mLN (**C**) 16 hours after photoconversion. Bar graph shows mean ± (SEM) of 6-8 animals combined from two to three independent experiments. One-way ANOVA with Tukey's multiple comparison post-test was used to compare statistical significance, *=p<0.05, **=p<0.01, ***=p<0.001, ****=p<0.0001

Figure S3

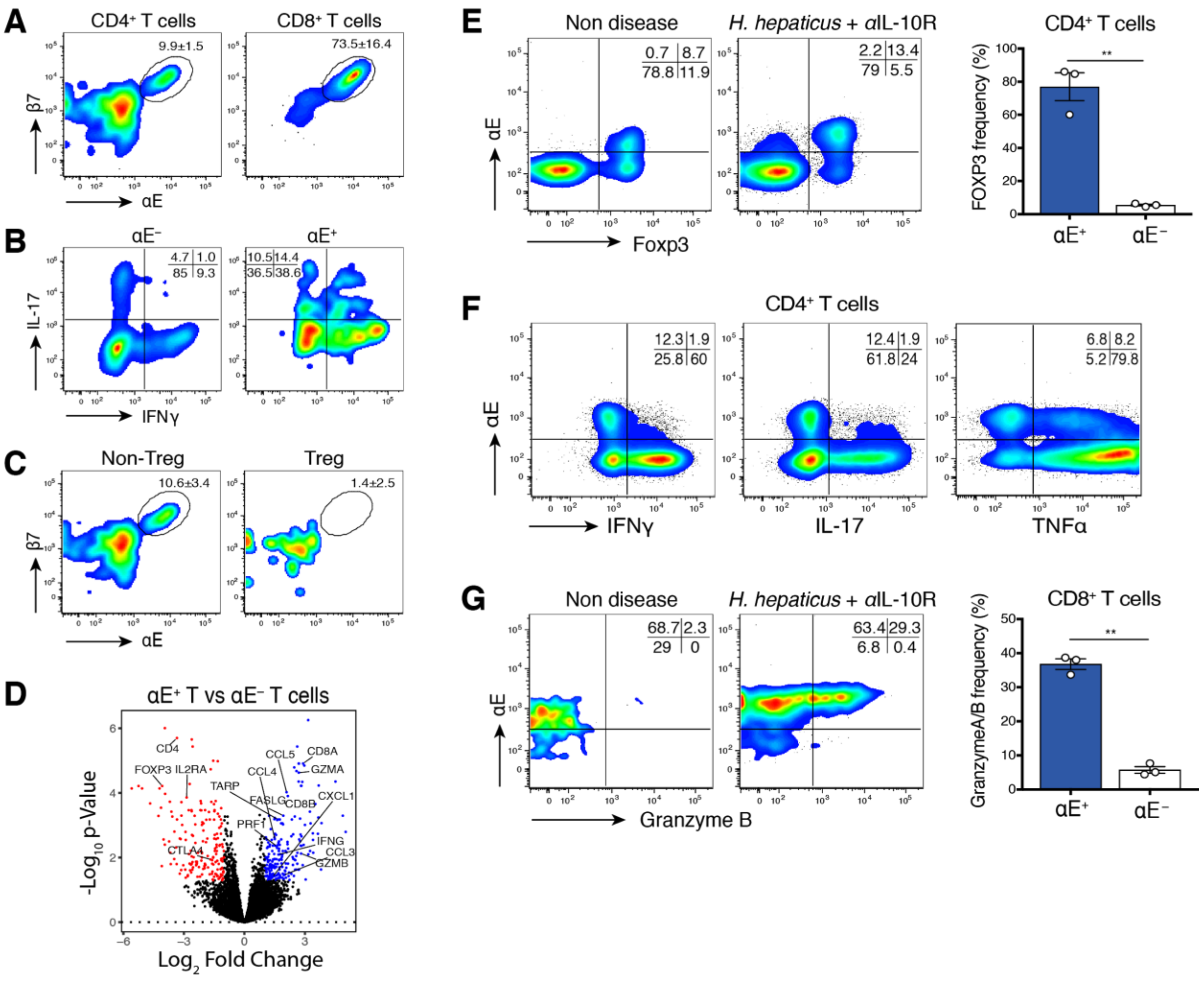

Figure S3. Proinflammatory state of αE$^+$ CD4$^+$ human T cells is not observed in mouse αEβ7$^+$ CD4$^+$ T cells. Related to Figure 5. T cells isolated from colonic tissue were evaluated for integrin expression and inflammatory phenotype. **(A)** αE and β7 expression on colonic CD4$^+$ and CD8$^+$ T cells. Data is shown from a representative healthy colonic biopsy. (**B**) *Ex vivo* stimulation of colonic T cells induced IL-17 and IFN-γ production cells in αE$^+$ or αE$^-$ CD4$^+$ T cells from a UC patient. (**C**) αE and β7 expression in colonic CD4$^+$Foxp3$^+$CD25$^+$CD127^{low} cells (Treg) and CD4$^+$ T cells from a healthy subject. (**D**) Volcano plot showing gene expression in sorted αE$^+$TCRβ$^+$ T cells vs αE$^-$ TCRβ$^+$ T cells from colonic resections (ulcerative colitis patients, n=4 and diverticulitis (non-IBD), n=4). (**E**) Similar phenotyping of mouse colonic T cells showing αE and Foxp3 expression in LP CD4$^+$ T cells isolated from naive or colitic mice treated with *H. hepaticus* and anti-IL-10R blockade (left). Right panel, frequency of Foxp3$^+$ regulatory T

cells among αE^+ $CD4^+$ T cells vs αE^- $CD4^+$ T cells isolated from colitic mice. Each dot represents an individual mouse and data is shown as mean ± SEM. (**F**) *Ex vivo* stimulation of colonic LP T cells from colitic mice treated with *H. hepaticus* and anti-IL-10R blockade induced intracellular cytokine expression. One representative flow cytometry pattern from four colitic animals in each group. (**G**) Left, αE and intracellular granzyme B expression in $CD8^+$ T cells isolated from colon LP in colitic mice treated with *H. hepaticus* and anti-IL-10R blockade. Right, frequency of granzyme A and B double positive cells among αE^+ or αE^- $CD8^+$ T cells from colitic mice. Each dot represents an individual mouse and data is shown as mean ± SEM. Data are representative of three independent experiments. Unpaired Student's t-test was used to compare statistical significance, *=$p<0.05$, **=$p<0.01$, ***=$p<0.001$, ****=$p<0.0001$

Figure S4

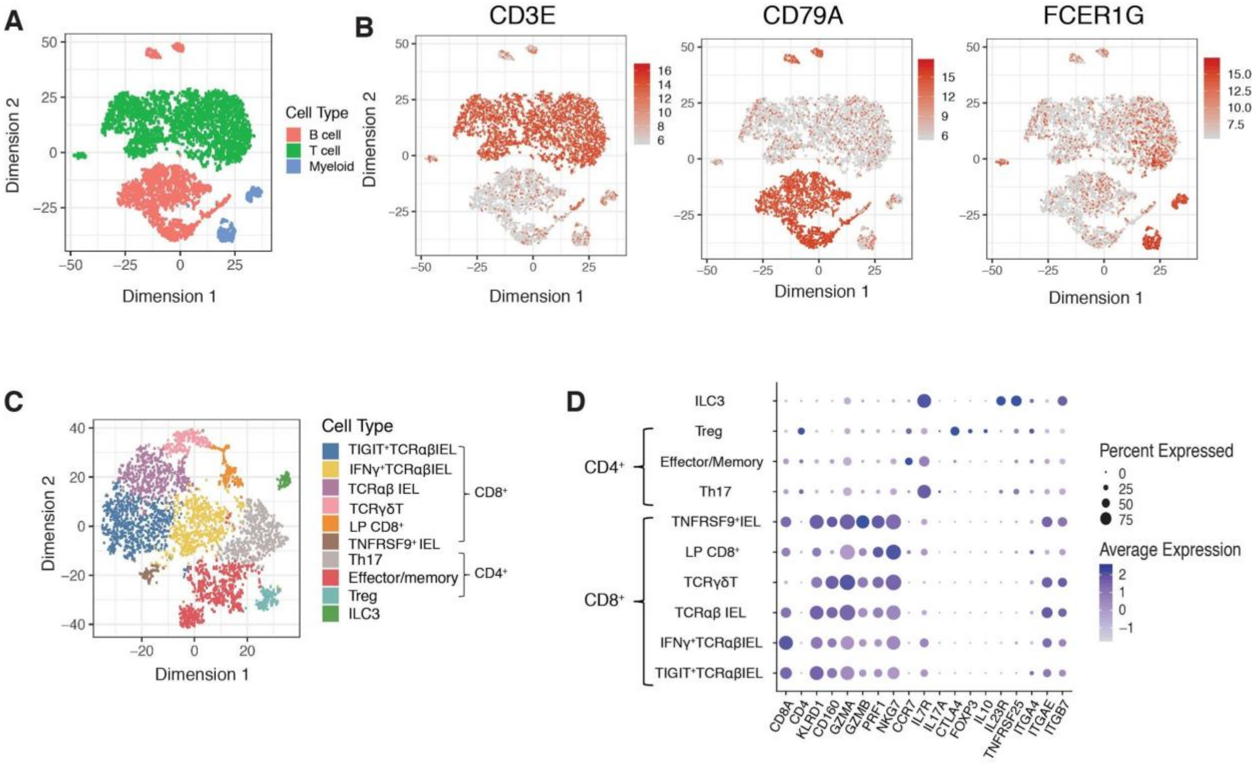

Figure S4. Clustering of human immune cells from colon. Related to Figure 5. (**A**) CD45^{+} immune cell census is shown as t-Stochastic Neighborhood Embedding (t-SNE) of cells colored by cell subsets. (**B**) Expression of CD3E, CD79A, and FCER1G in the corresponding cell population. (**C**) T cell census is shown as t-SNE of cells colored by cell subsets. (**D**) T cell subsets and their markers. Shown are fractions of expression (dot size) and mean expression level in expressing cells (dot color) of selected marker genes (columns) across subsets (rows).

Figure S5

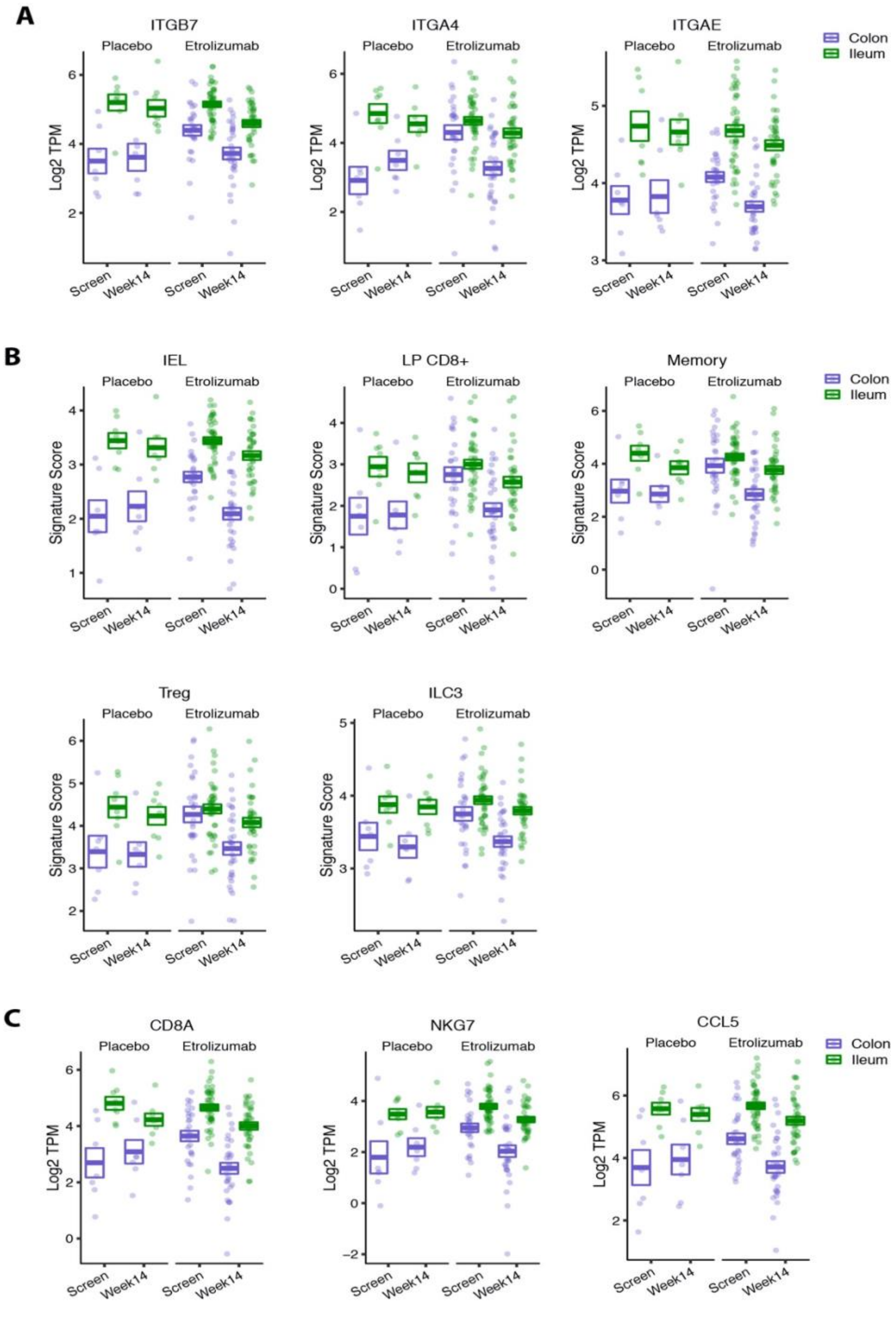

Figure S5. Etrolizumab treatment significantly reduces expression of genes associated with CD8⁺ cytotoxic IELs. Related to Figure 6. Ileal or colonic biopsies were taken prior to treatment and at 14 weeks post-treatment in

a randomized placebo-controlled trial of etrolizumab (anti-β7 integrin) in patients with moderately to severely active CD (A) Expression of integrin genes at 14 weeks post-etrolizumab or placebo treatment. Each dot is an individual patient. Points are colored by sampling location (colon in blue, ileum in green). Boxes show the standard error, with the middle bar showing the group mean value. (B, C) Expression of the signature gene sets of indicated T cell subtype gene sets (B) and the cytotoxic IEL-specific genes (C) at week 14 in patients treated with etrolizumab or placebo. Each dot represents an individual patient sample. Points are colored by sampling location (colon in blue, ileum in green). Boxes show the standard error, with the middle bar showing the group mean value.

Figure S6

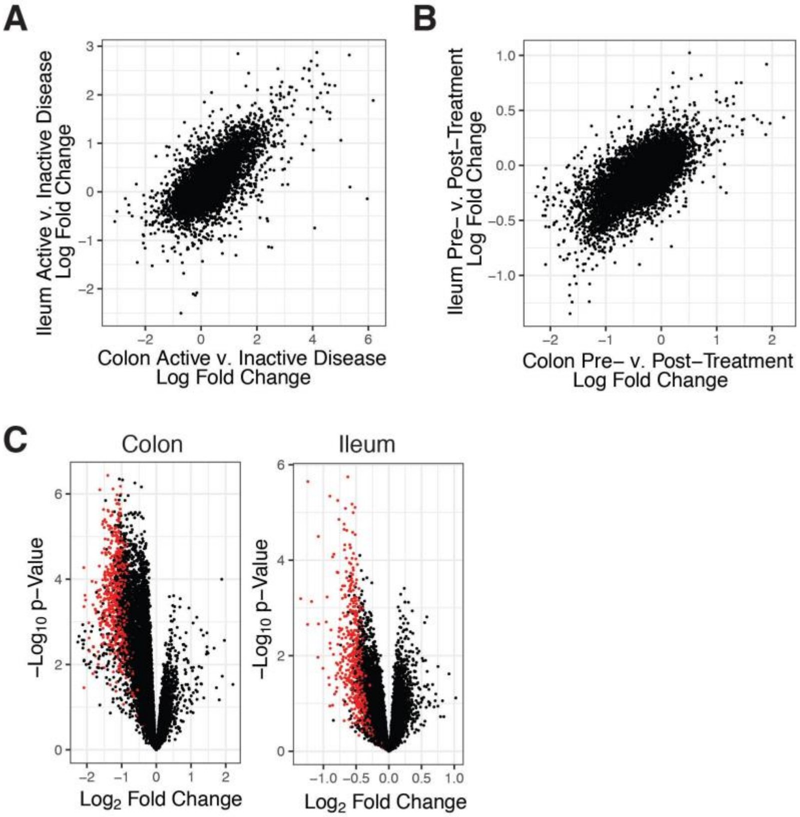

Figure S6. Etrolizumab treatment reduces inflammatory gene expression in both the colon and the ileum. Related to Figure 6. Scatter plots showing the log2 fold changes between samples taken from bowel segments with active vs inactive disease in either colon or ileum (**A**) or the log2 fold changes between samples taken from post-induction vs screening samples from etrolizumab-treated patients in either colon or ileum (**B**). (**C**) Volcano plot showing the log2 fold change and p-value of each gene comparing baseline to week 14 samples in etrolizumab-treated patients in either colon (left panel) or terminal ileum (right panel). Each point represents a gene, genes with a >1.5 fold change at an FDR < 0.05 in the pooled analysis combining the colon and ileum are shown in red.

Figure S7

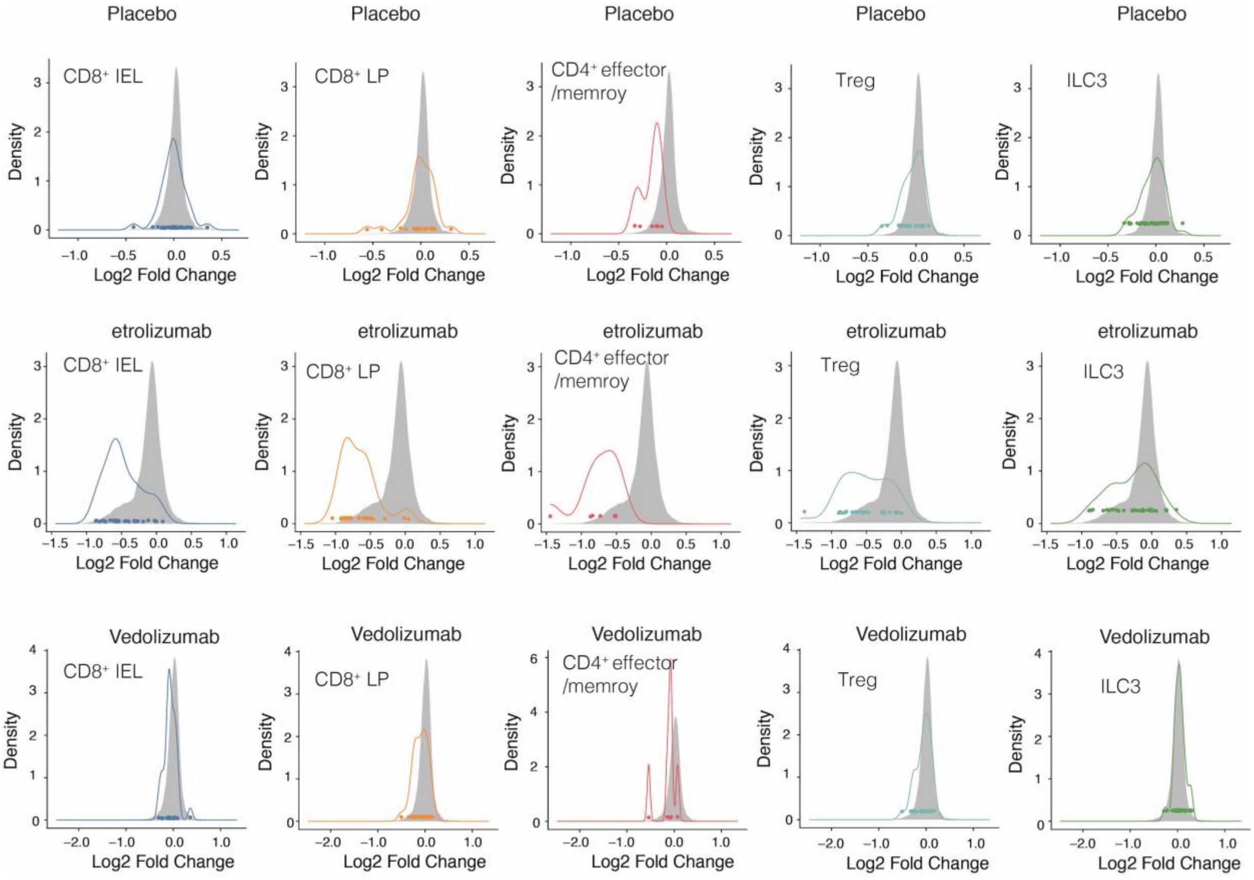

Figure S7. Etrolizumab, but not Vedolizumab or placebo, significantly reduces the gene set associated with CD8⁺ IELs and CD8⁺ LP populations. Related to Figure 6. Density plots of the slope of change in gene expression between baseline and post-treatment biopsies cohorts for each gene. Etrolizumab and placebo post-treatment biopsies were taken at week 14, while vedolizumab post-treatment biopsies were taken at week 12. The background distribution of all fold changes is shown in grey and the individual fold changes for genes in each T cell signature found to be downregulated in the placebo, vedolizumab or etrolizumab-treated patients are shown with solid points. The density distribution of these points are shown with solid lines.

Supplemental file 1. Heatmap of genes specific to each T cell subset. Each row is a gene, each column is a cell. T cell types are indicated across the top x-axis. Related to Figure 5.

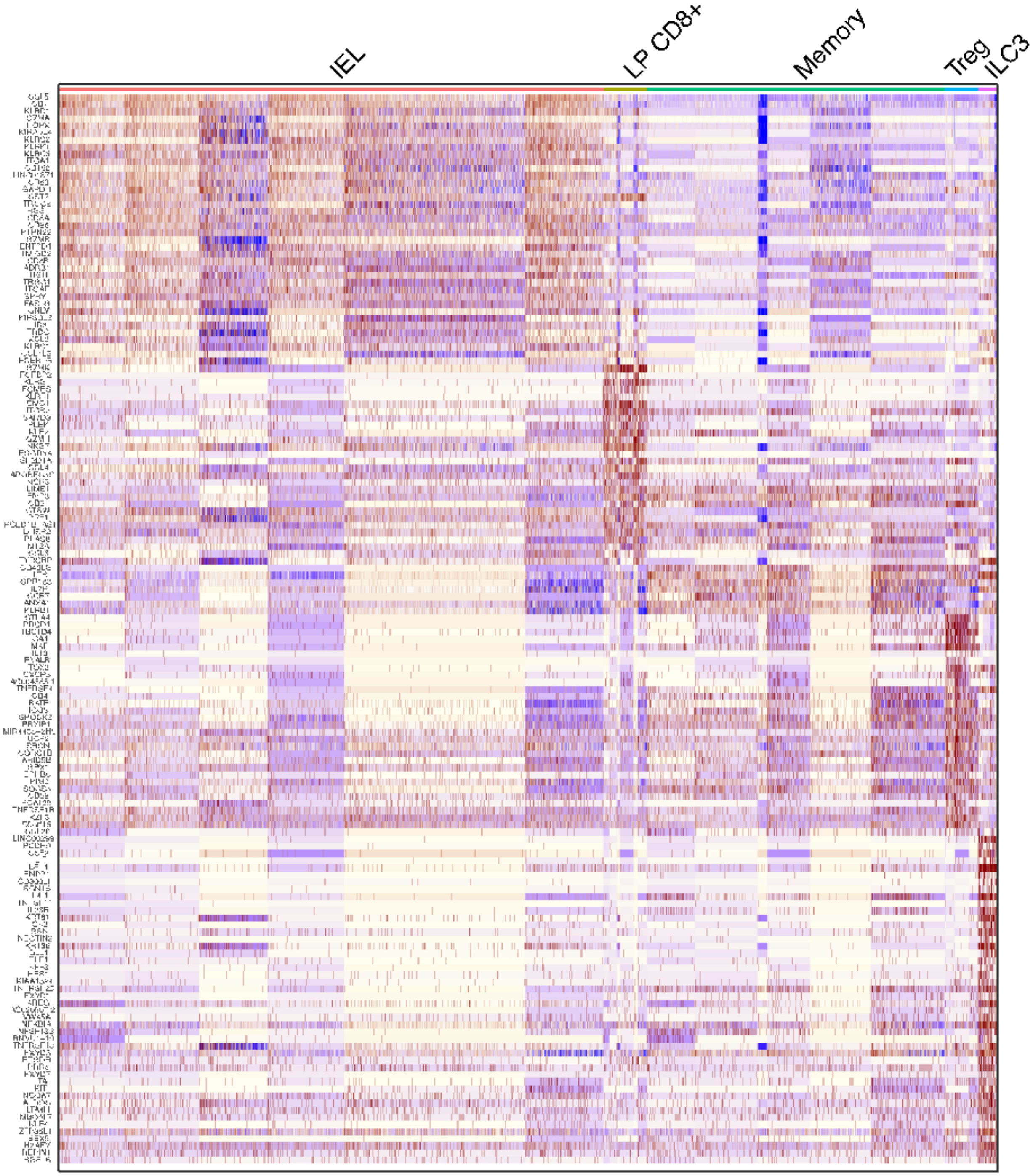

Table S1. Patient characteristics from observational cohorts. Related to Figure 5.

Cohort	Gender	Age	Diagnosis
Mayo Clinic	F/F	59	diverticulitis
	F/F	44	UC
	M/M	23	UC
	M/M	25	UC
	F/F	35	UC
	M/M	58	diverticulitis
	F/F	68	diverticulitis
	M/M	37	UC
Stanford	M/M	65	Healthy control
	F/F	58	UC
	F/F	56	UC
	F/F	42	CD
	F/F	38	CD
UCSF	F/F	32	Healthy control
	M/M	55	Healthy control
	F/F	34	Healthy control
	M/M	62	Healthy control
	M/M	38	Healthy control
	F/F	56	Healthy control
	M/M	26	Healthy control
	M/M	47	Healthy control
	M/M	68	Healthy control
	M/M	39	Healthy control

Table S2. Summary statistics for moderate to severe CD patients enrolled in etrolizumab clinical study with RNA sequencing data. Related to Figure 6.

**All subjects**	**Placebo (n=45)**		**Etrolizumab (n=172)**	
Biopsy location	colon (n=22)	ileum (n=23)	colon (n=70)	ileum (n=102)
Gender, female/male	9F / 13M	15F / 8M	35F / 35M	54F / 48M
Age in years, median (range)	31.5 (18-63)	39.0 (24-73)	35.0 (19-79)	42.0 (19-67)
SES-CD, median (range)	19.5 (6-44)	17.0 (5-35)	21.0 (9-38)	15.0 (5-50)
Disease extent Colonic (C), colonic+ileal (IC), ileal (I)	11C, 9IC, 2I	2C, 15IC, 6I	36C, 30IC, 4I	9C, 60IC, 33I
**Subjects with paired measures of active disease segments**	**Placebo (n=15)**		**Etrolizumab (n=71)**	
Biopsy location	colon (n=7)	ileum (n=8)	colon (n=28)	ileum (n=43)
Gender, female/male	6F / 1M	5F / 3M	18F / 10M	28F/15M
Age in years, median (range)	38.0 (27-51)	36.5 (24-62)	38.0 (19-79)	42.0 (19-67)
SES-CD, median (range)	19.0 (15-44)	14.5 (7-35)	21.0 (11-37)	15.0 (7-44)
Disease extent Colonic (C), colonic+ileal (IC), ileal (I)	4C, 3IC, 0I	0C, 5IC, 3I	16C, 12IC, 0I	0C, 27IC, 16I
